# Supplementary material for: Inhibition of IL-11 signalling extends mammalian healthspan and lifespan
Source: Nature. 2024 Jul 17;632(8023):157–65. doi: 10.1038/s41586-024-07701-9 (PMC11291288; doi:10.1038/s41586-024-07701-9)

nature portfolio

https://doi.org/10.1038/s41586-024-07701-9

Supplementary information

Inhibition of IL-11 signalling extends mammalian healthspan and lifespan

In the format provided by the authors and unedited

Supplementary Figure 1. Raw blots

Figure 1b and Extended Data Figure 1a

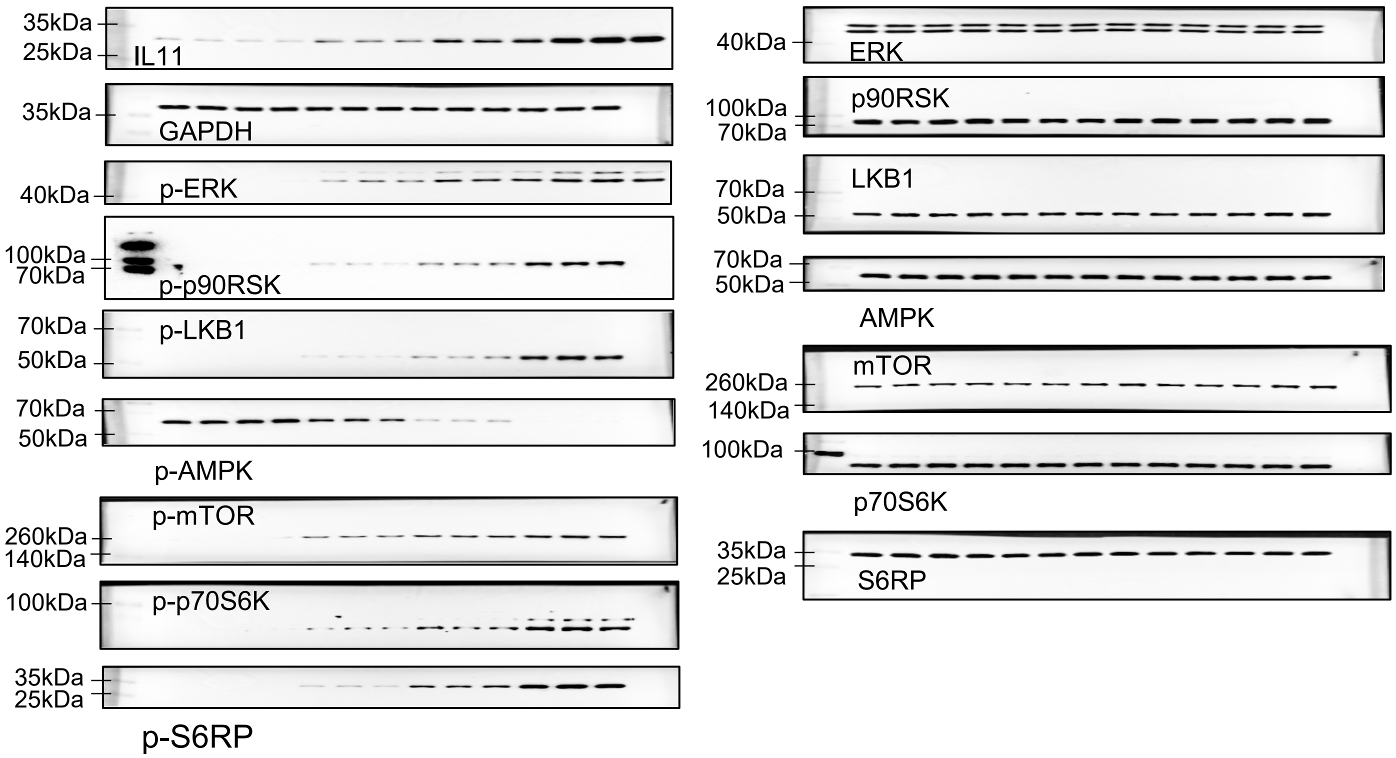

Figure 1c and Extended Data Figure 1b

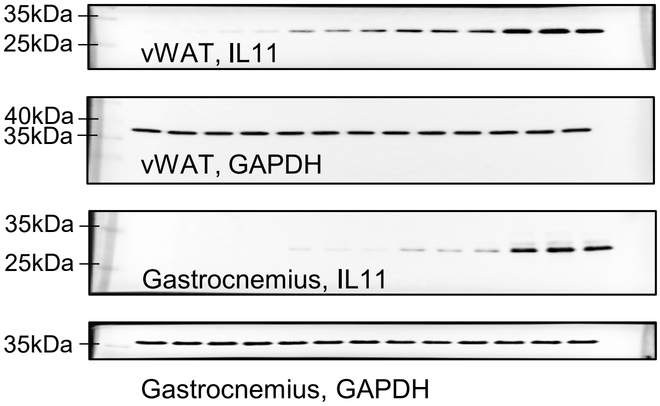

Figure 1e and Extended Data Figure 2a

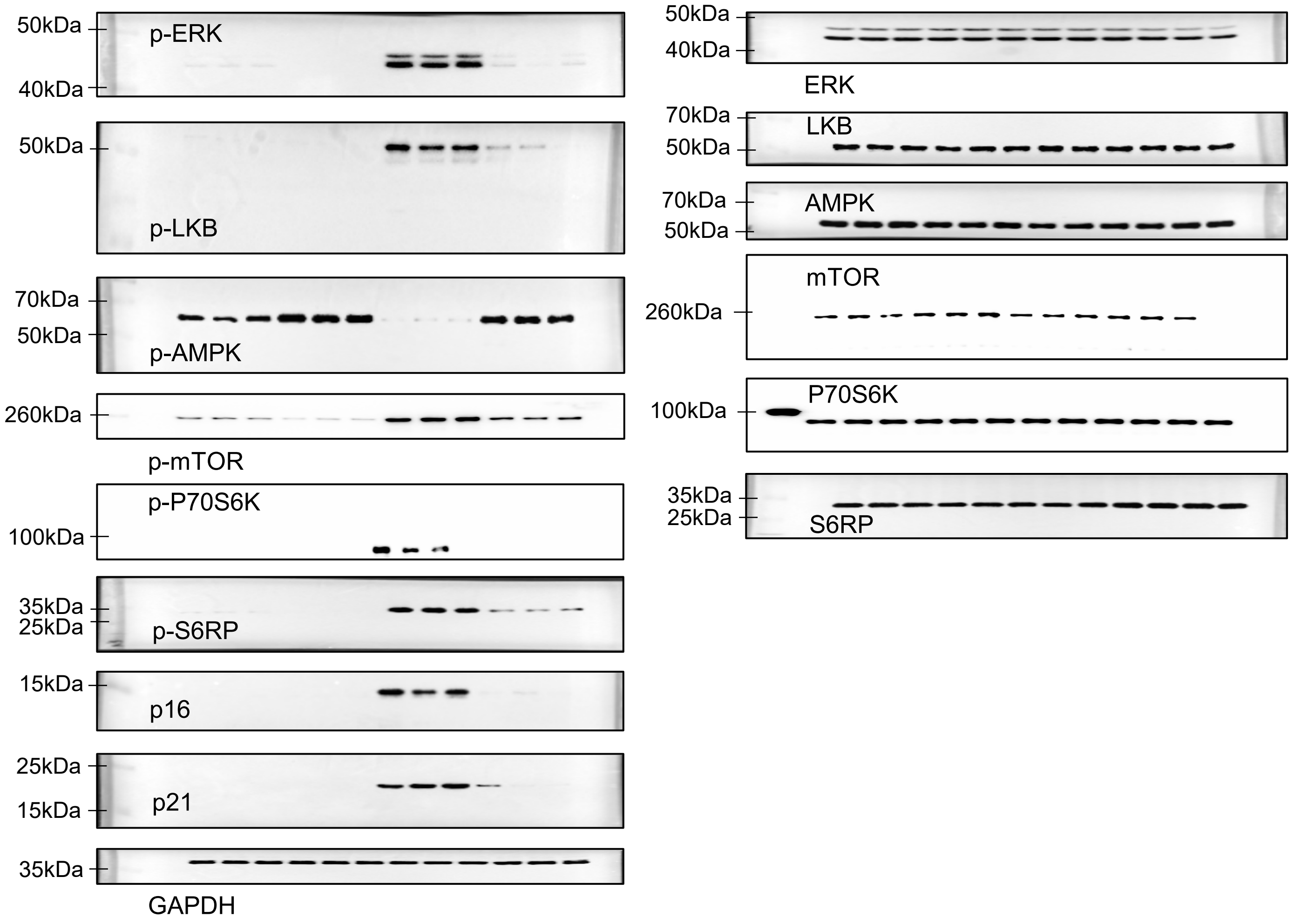

Figure 1j

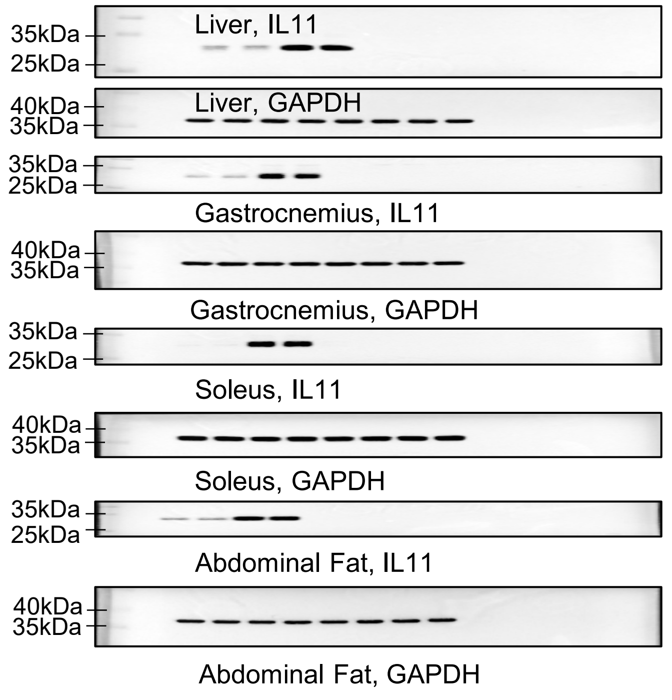

Figure 2j and Extended Data Figure 5k

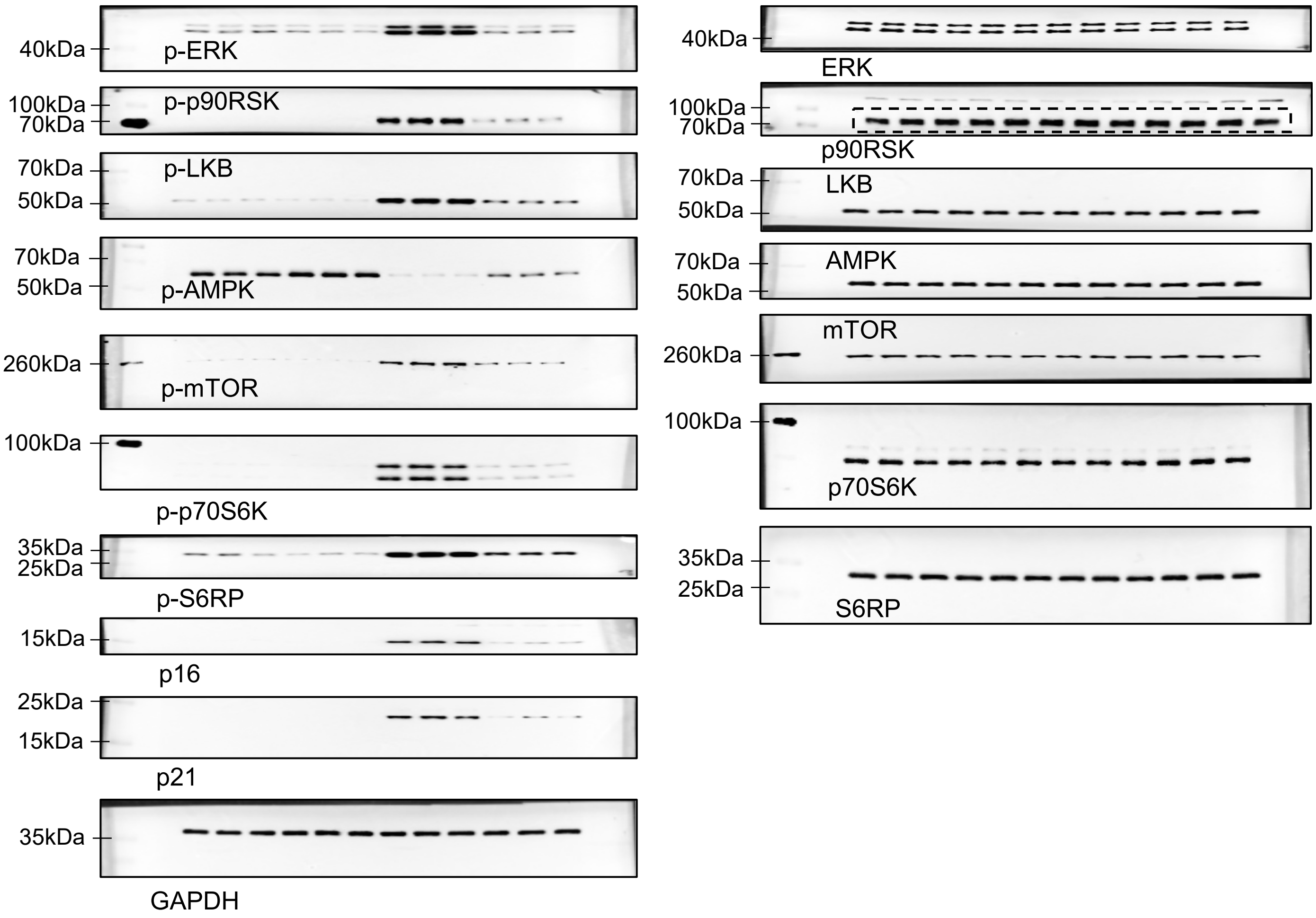

Figure 3m and Extended Data Figure 7i

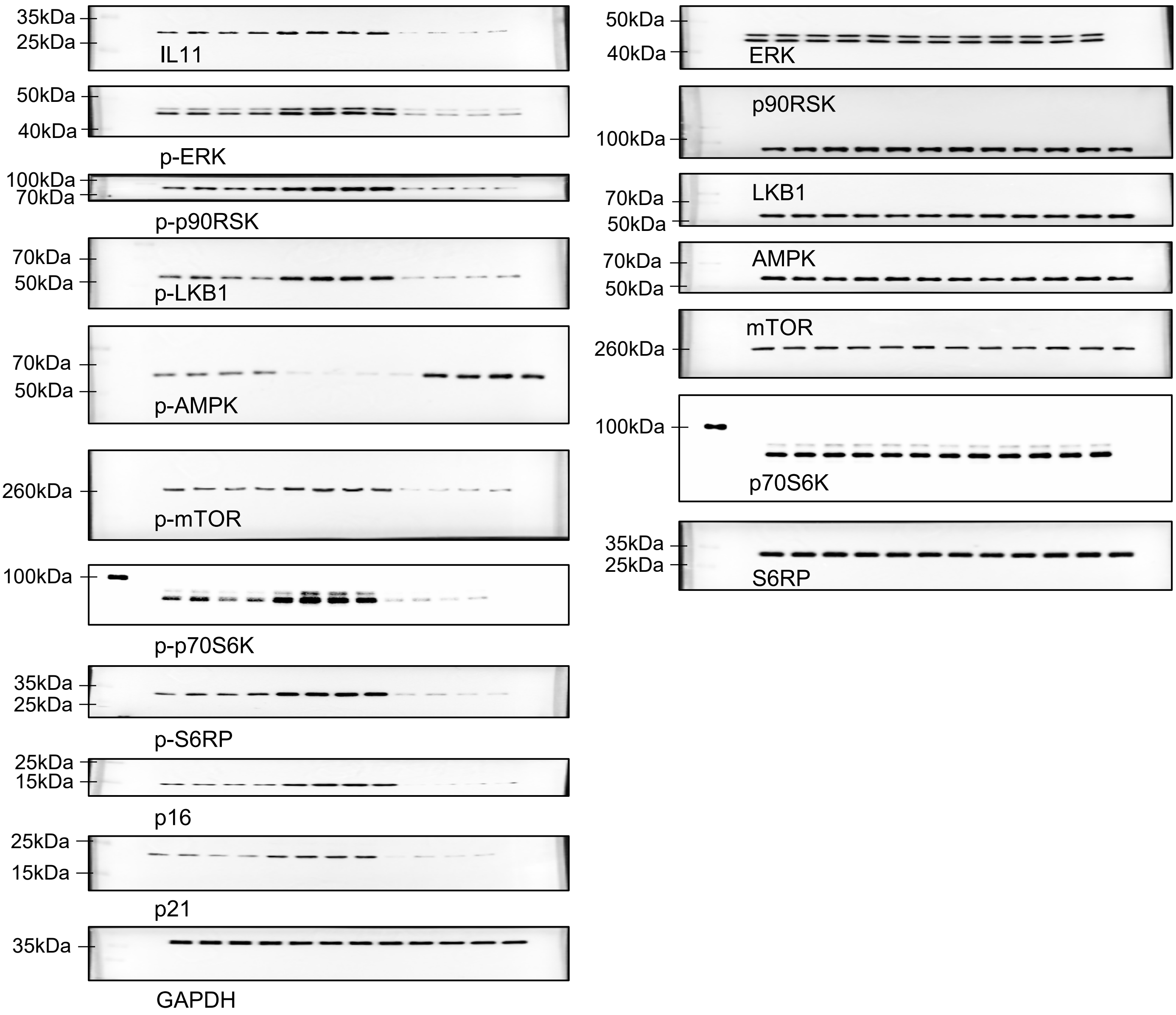

Figure 4e

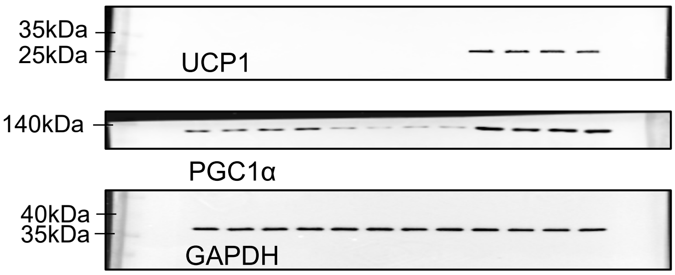

Figure 4f

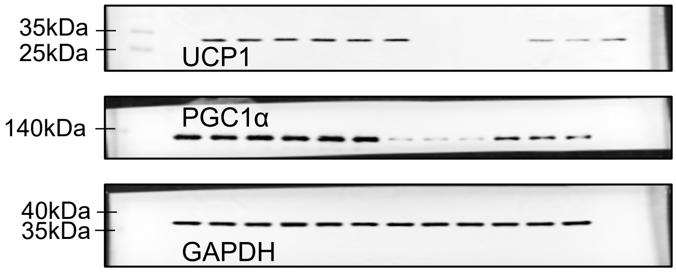

Extended Data Figure 1b

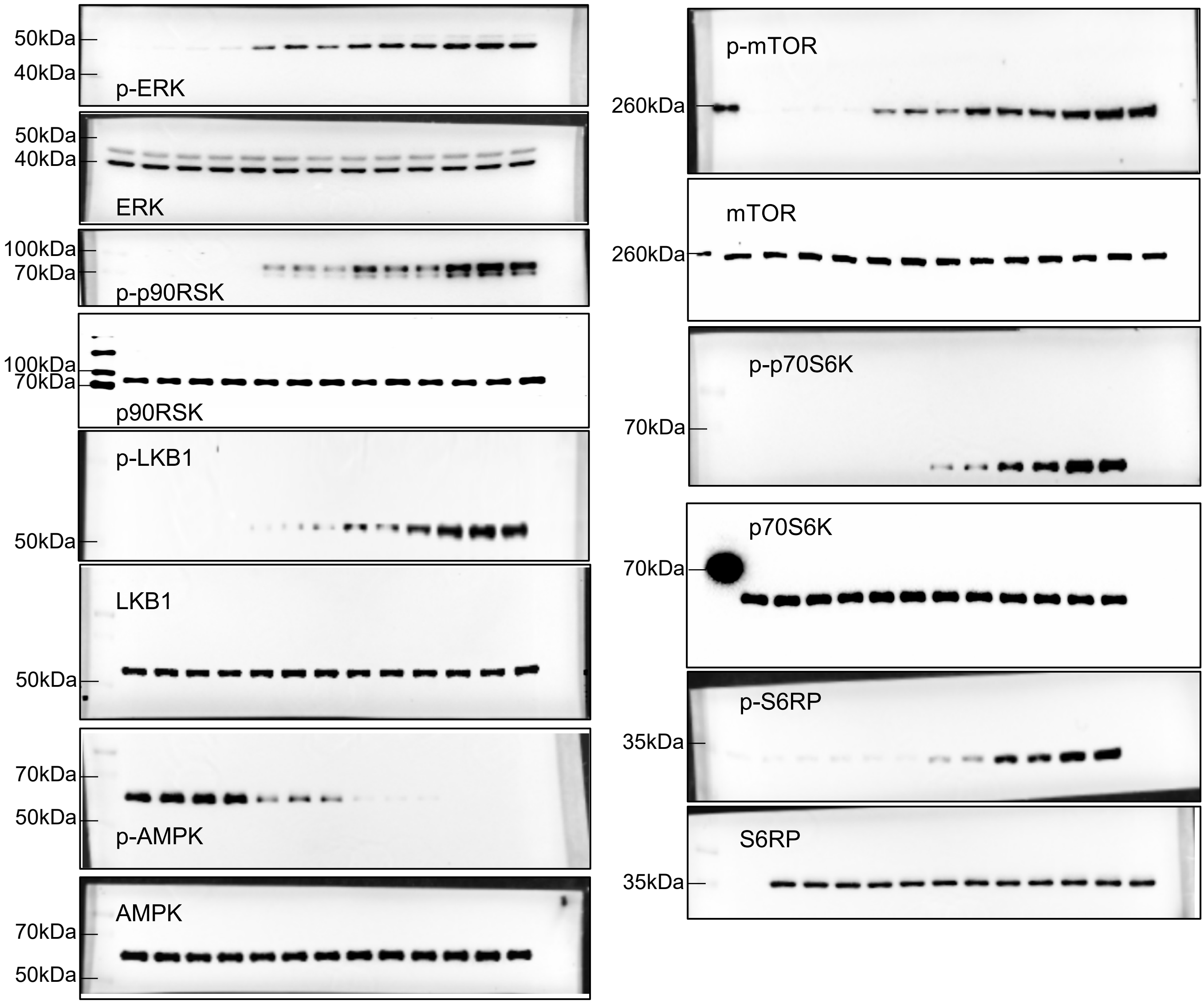

Extended Data Figure 1d

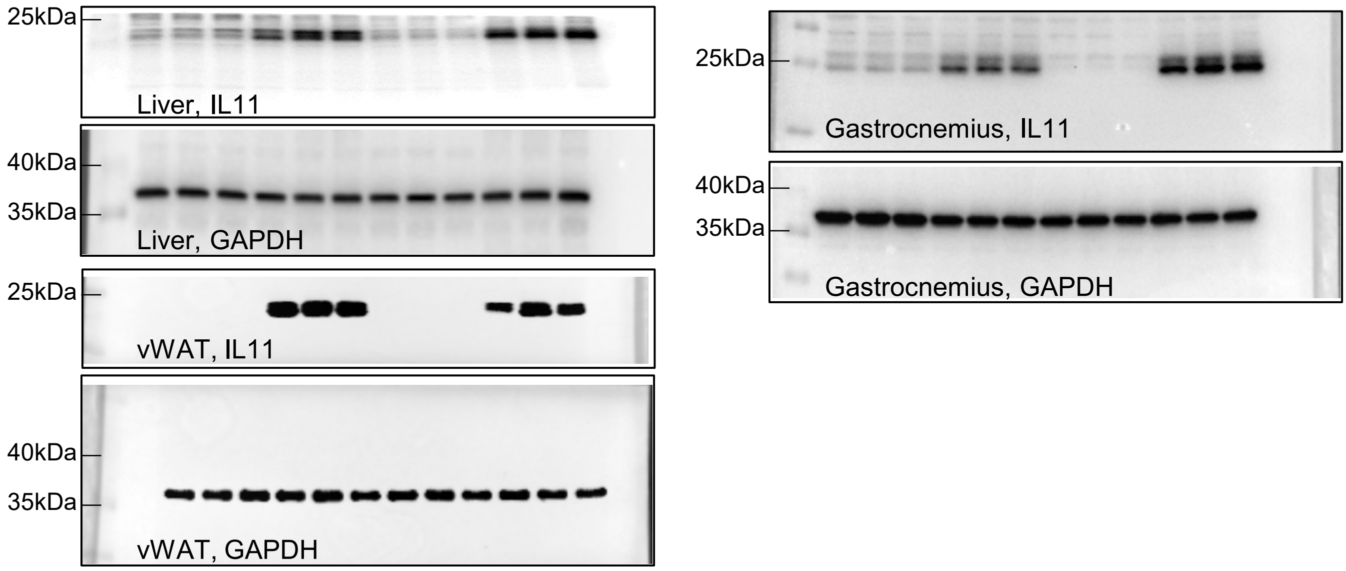

Extended Data Figure 2b

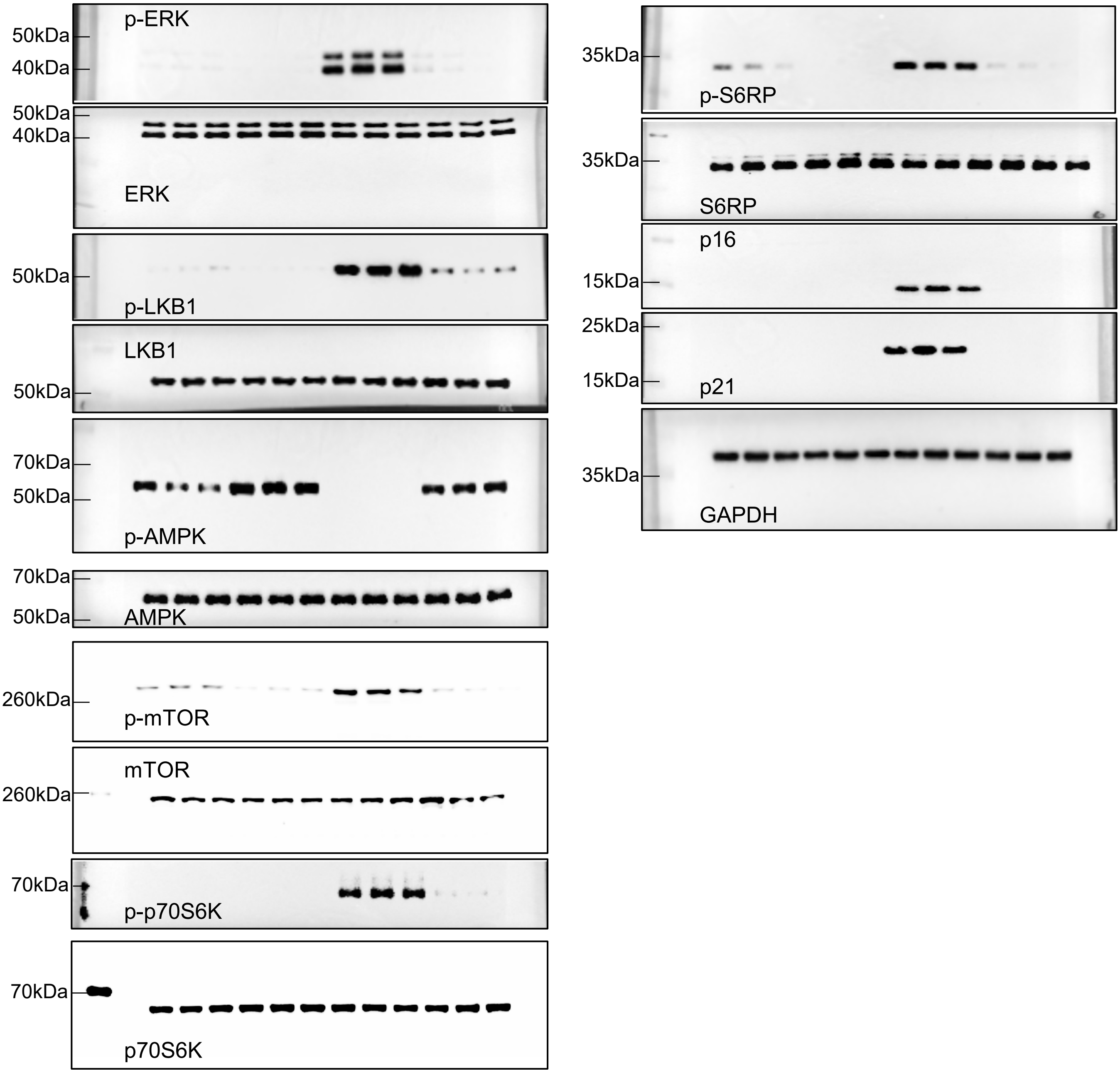

Extended Data Figure 2c

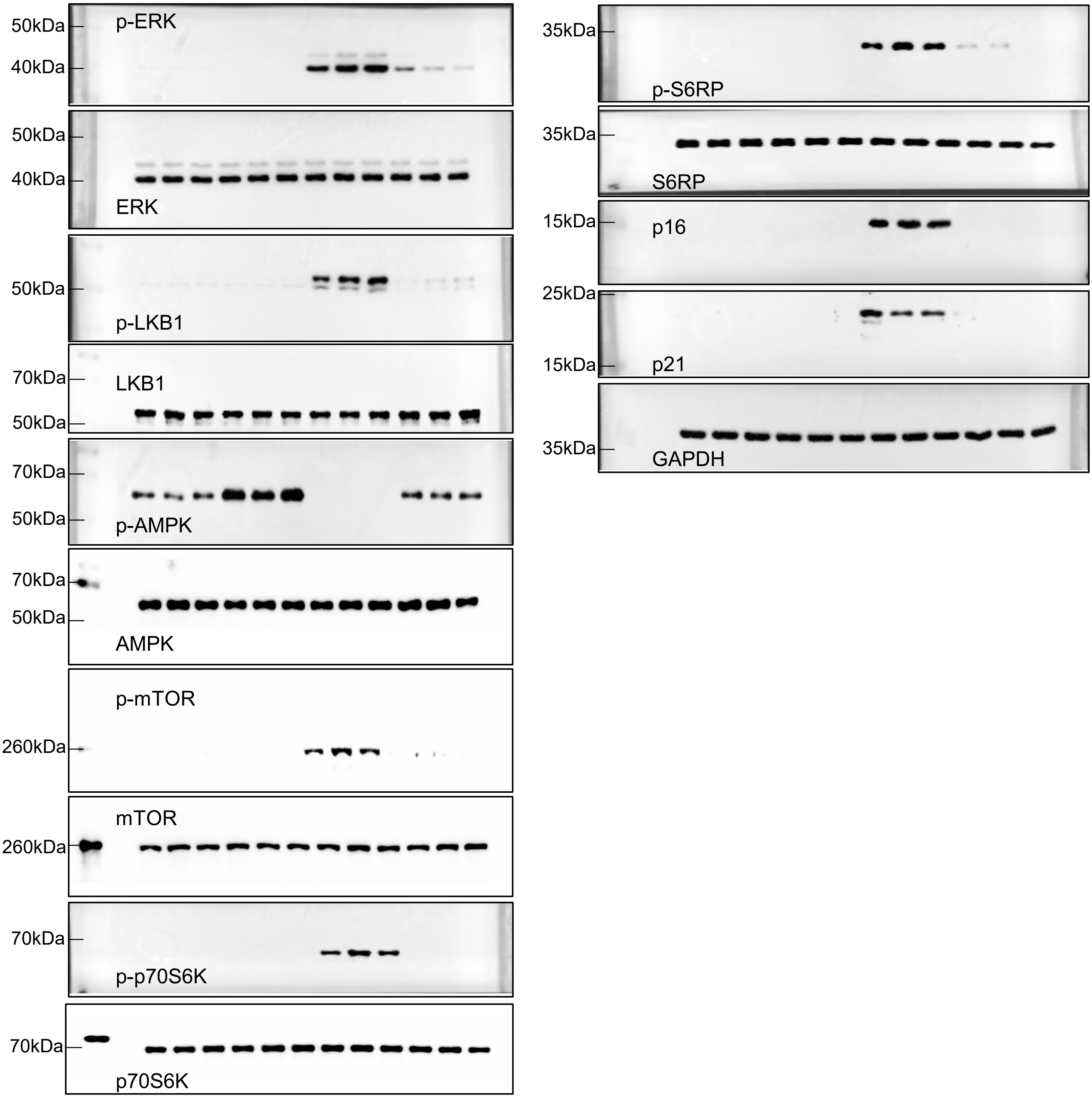

Extended Data Figure 3a

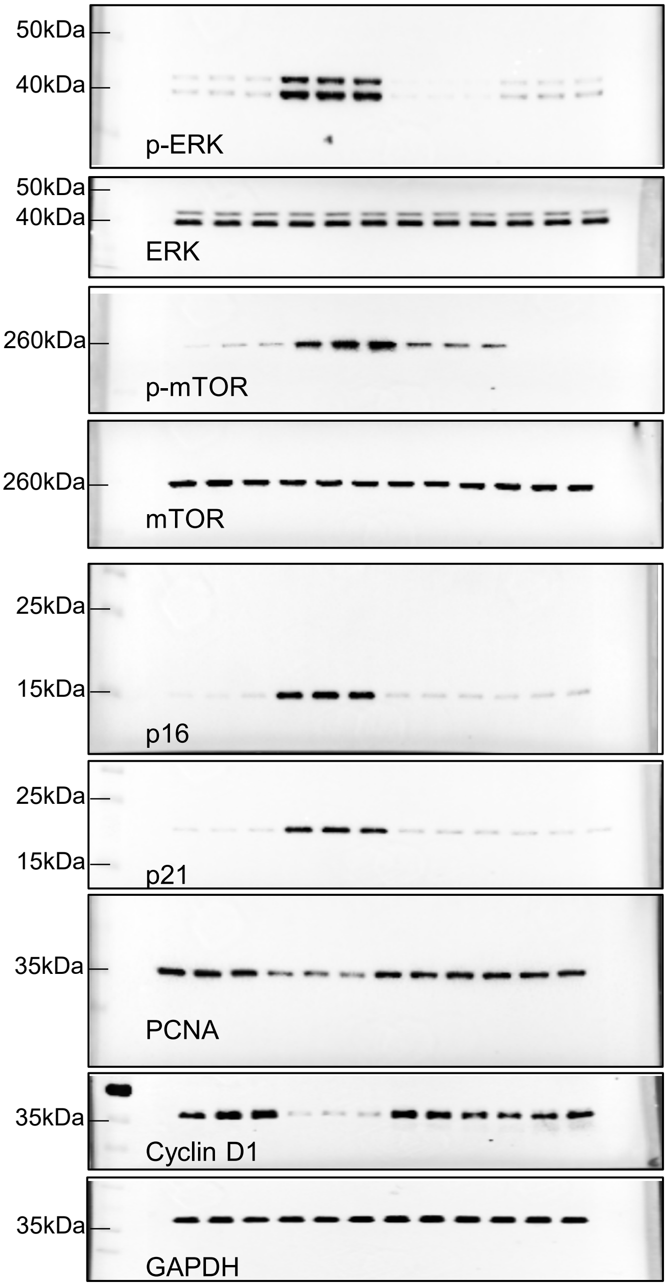

Extended Data Figure 3b

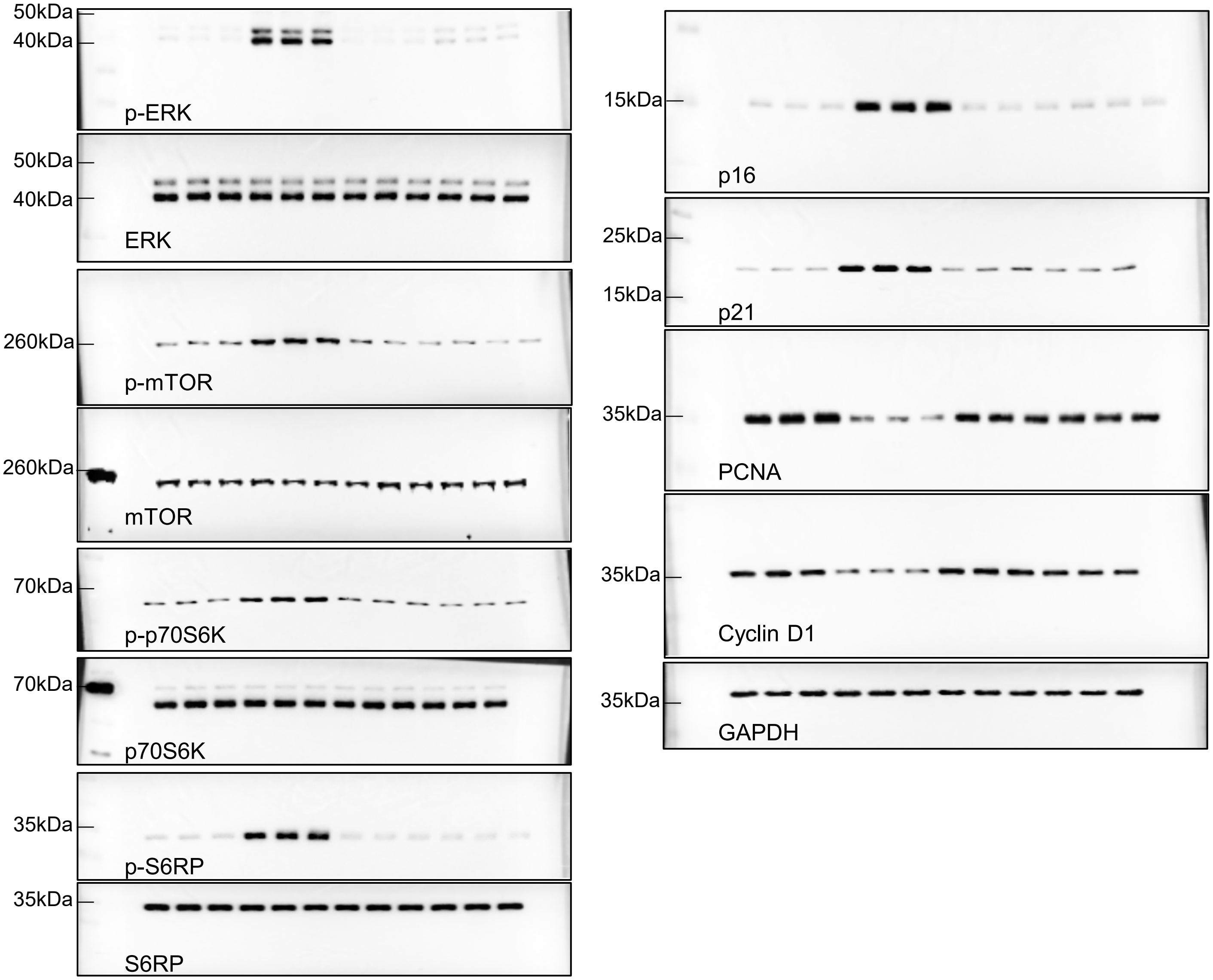

Extended Data Figure 4a

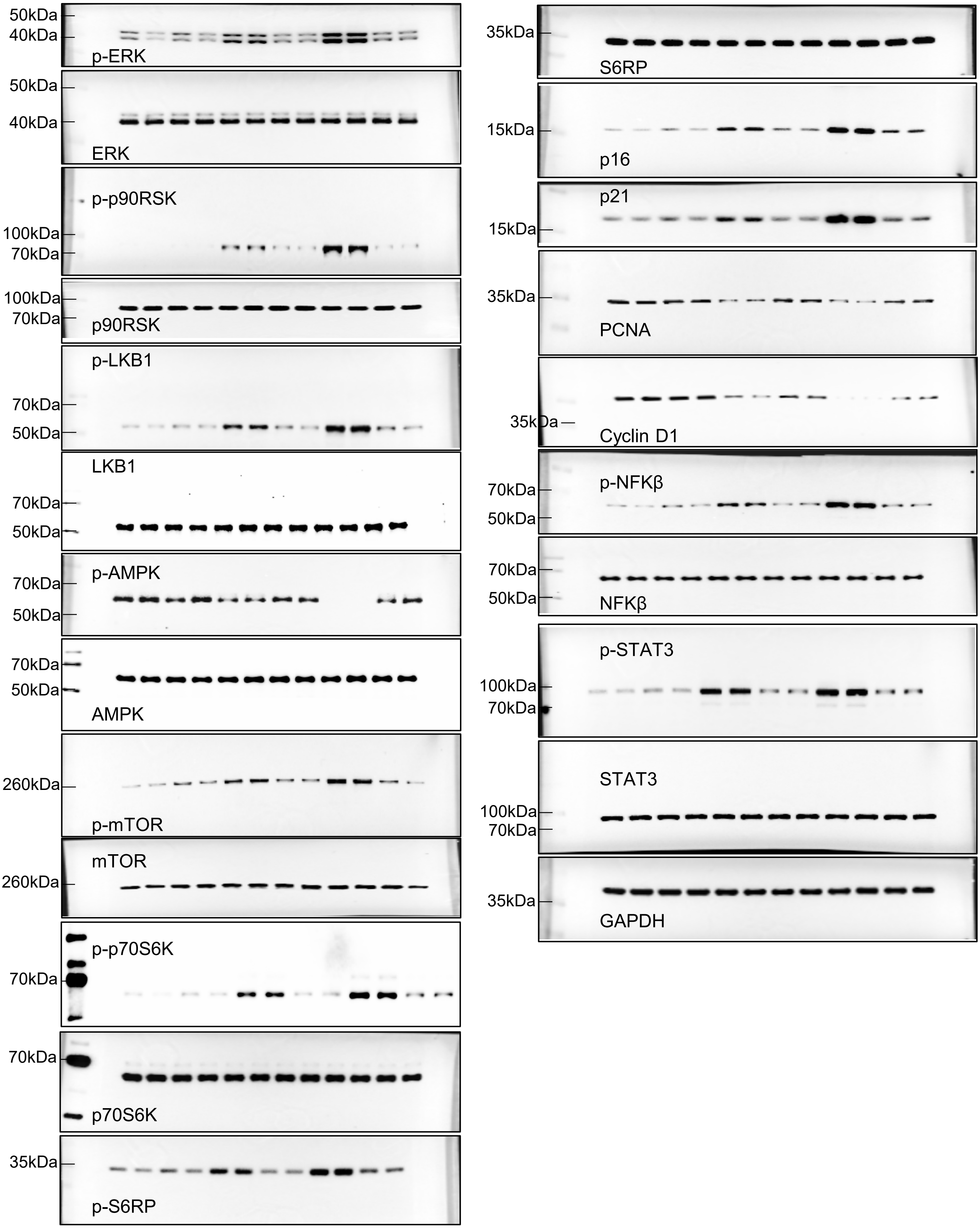

Extended Data Figure 4d

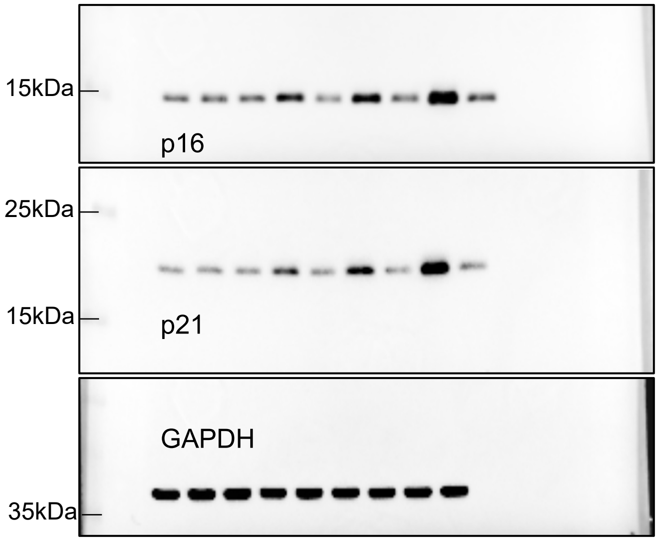

Extended Data Figure 5l

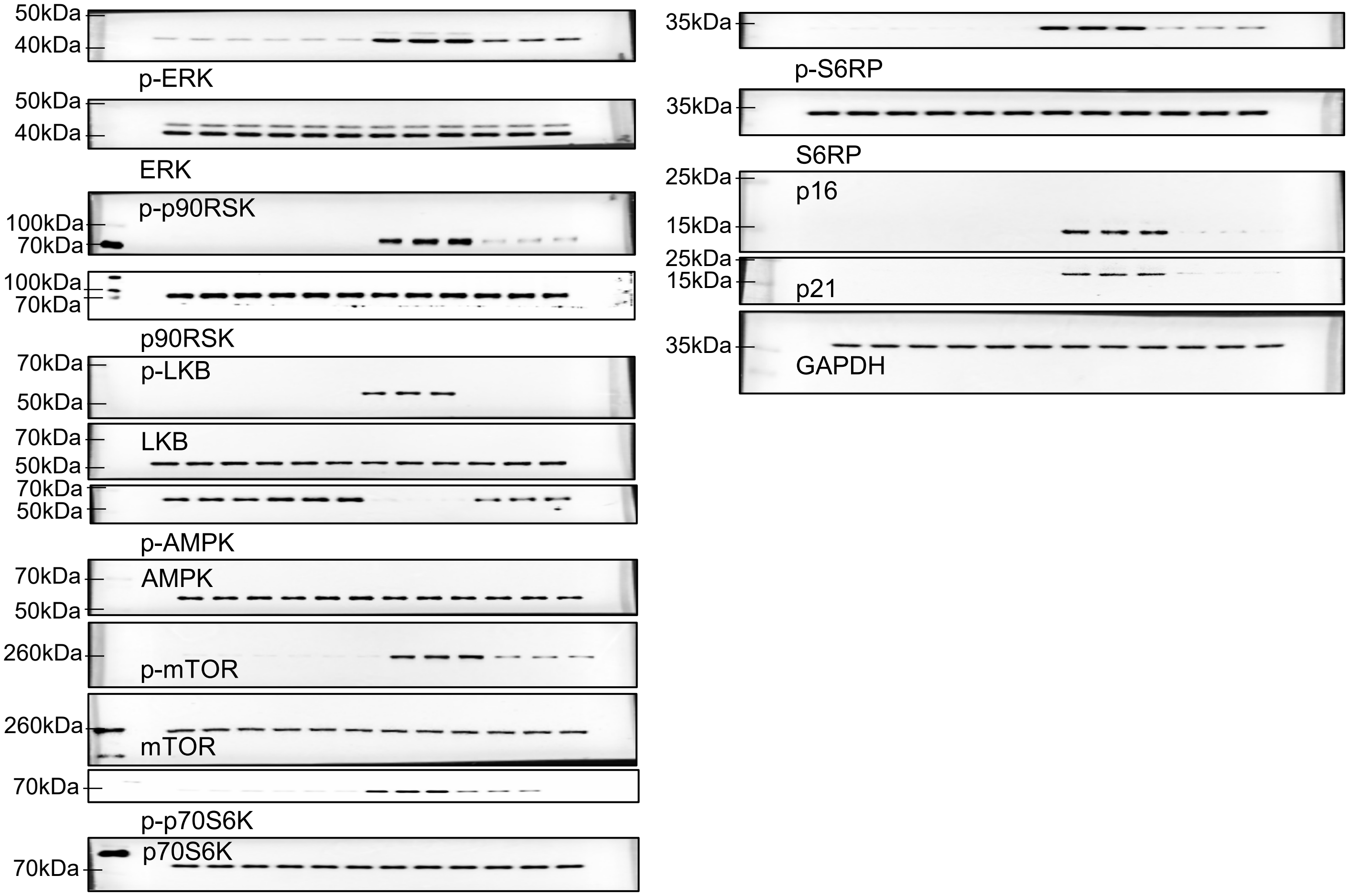

Supplementary Figure 2. Immunoblots densitometry analysis.

Figure 1b (total protein blots in Extended Data Figure 1a)

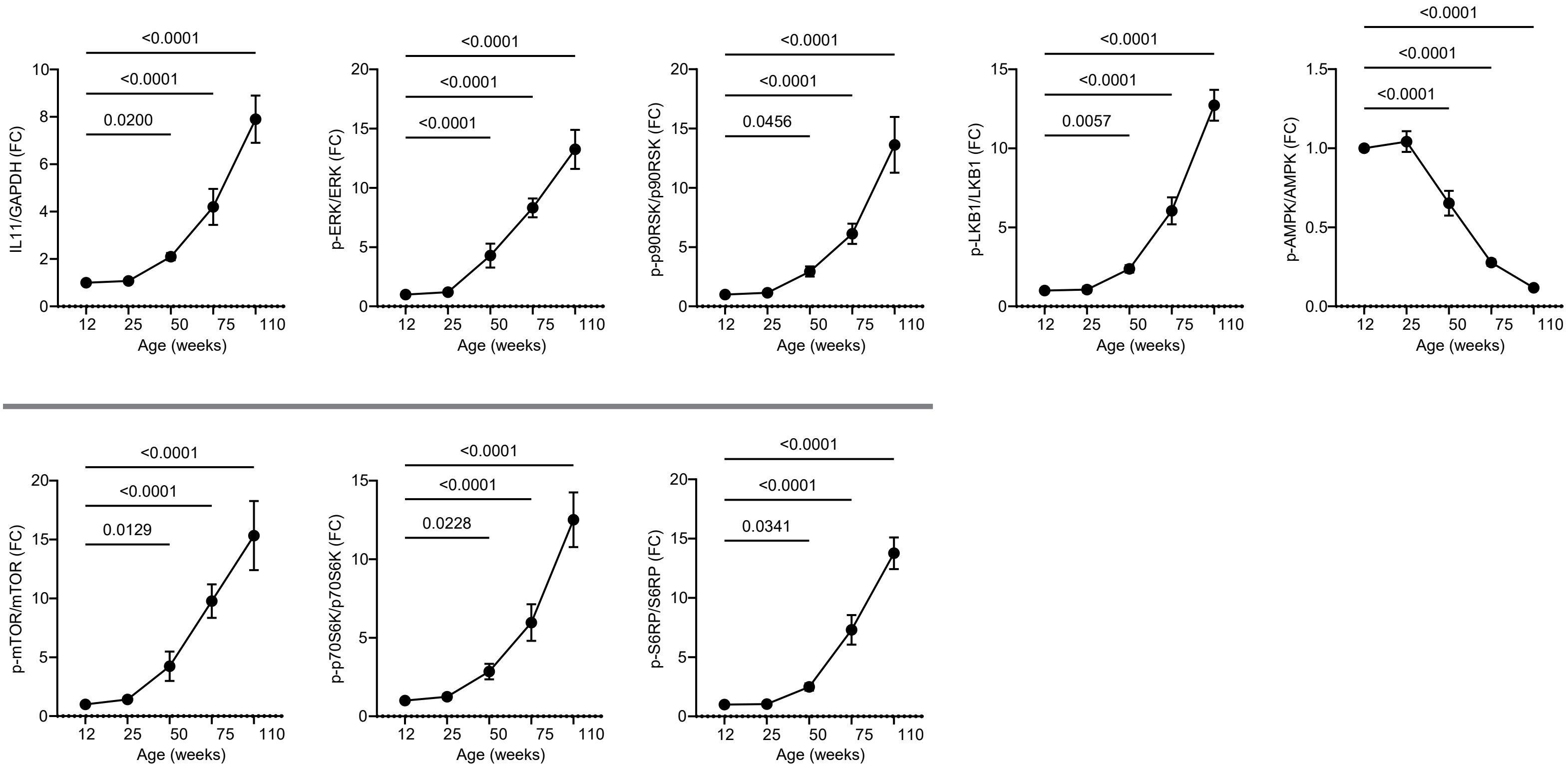

Figure 1e (total protein blots in Extended Data Figure 2a)

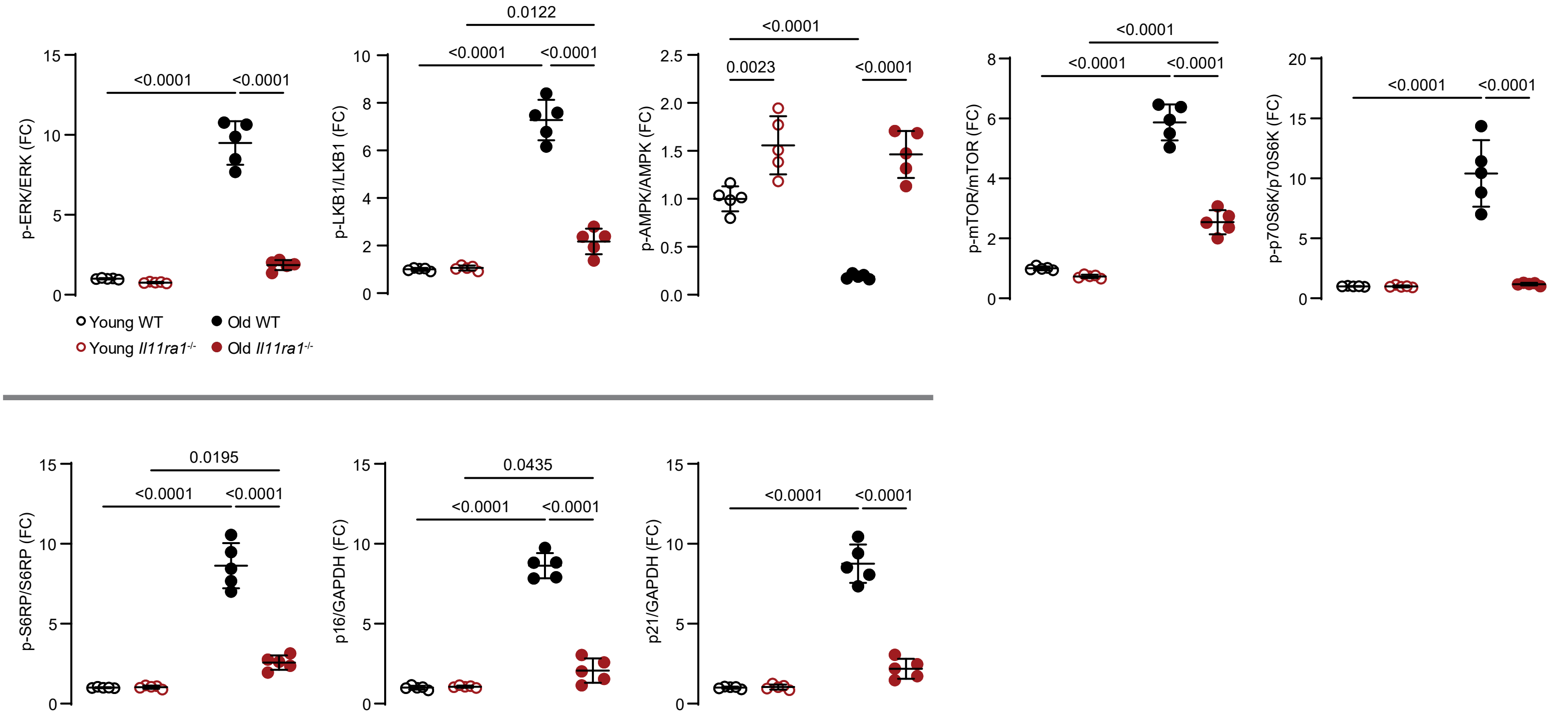

Figure 1j

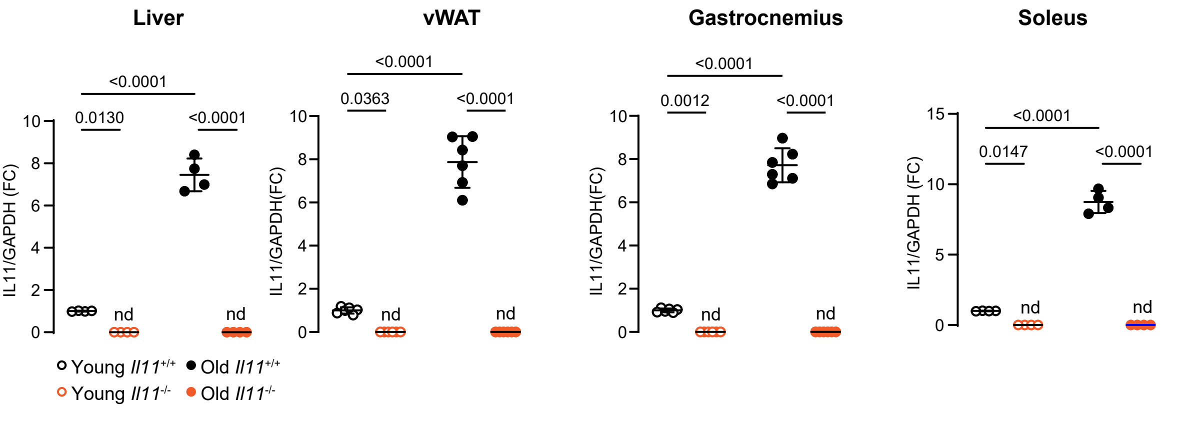

Figure 2j (total protein blots in Extended Data Figure 5k)

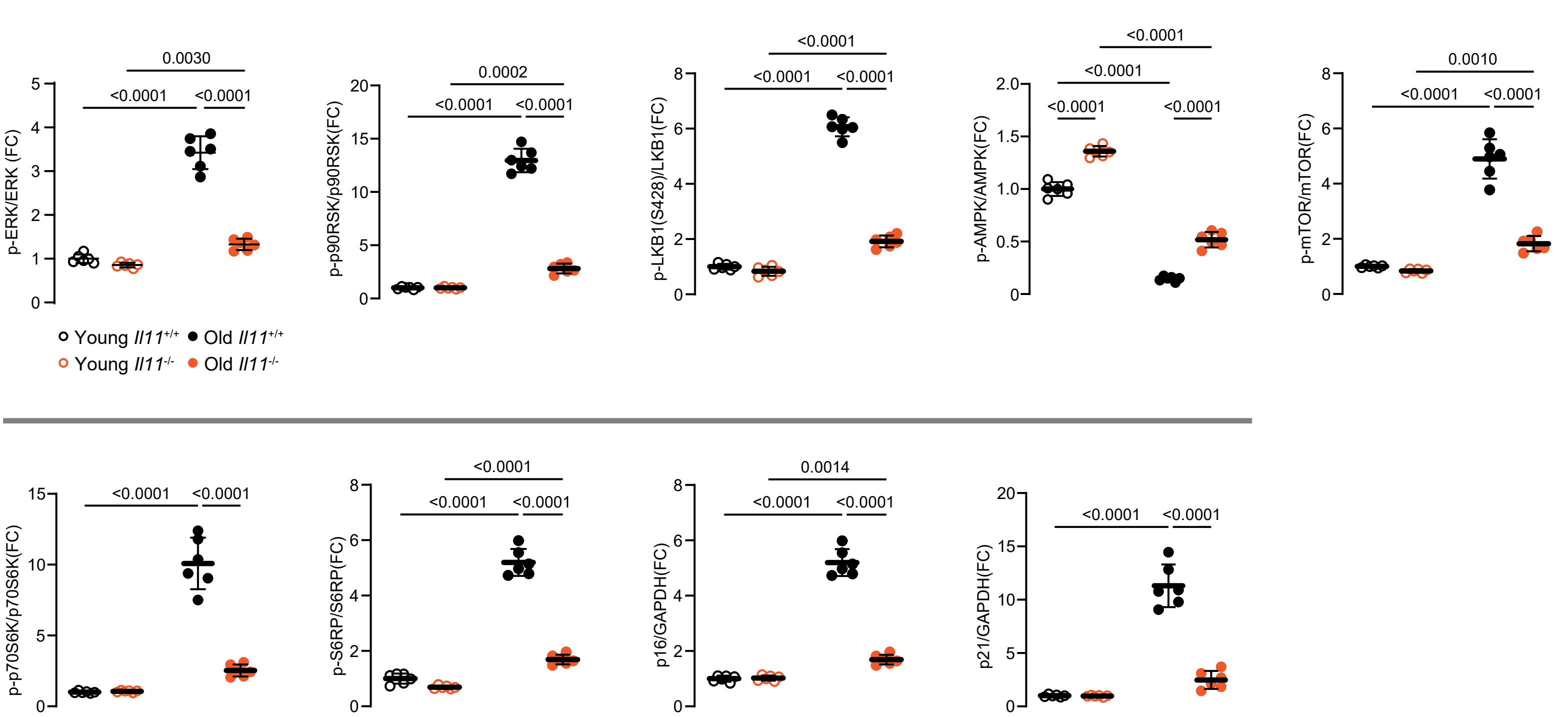

Figure 3m (total protein blots in Extended Data Figure 7i)

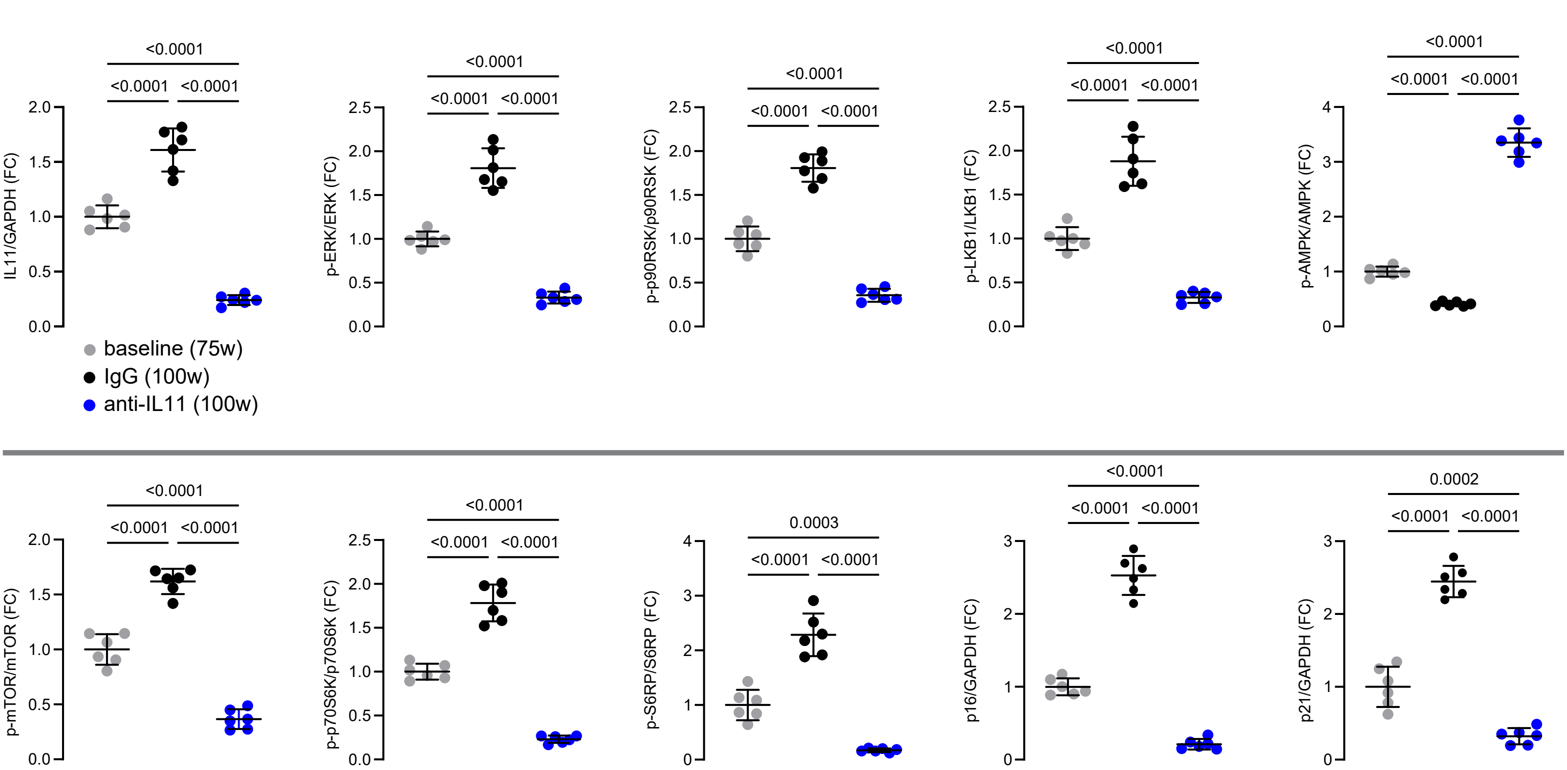

Figure 4e

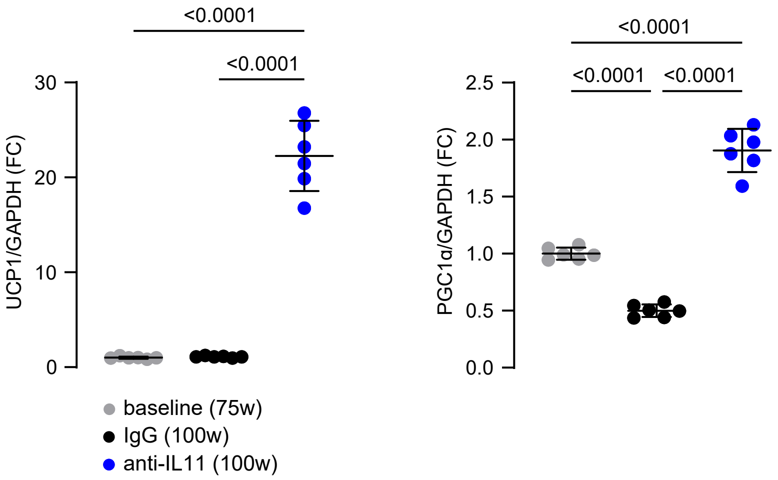

Figure 4f

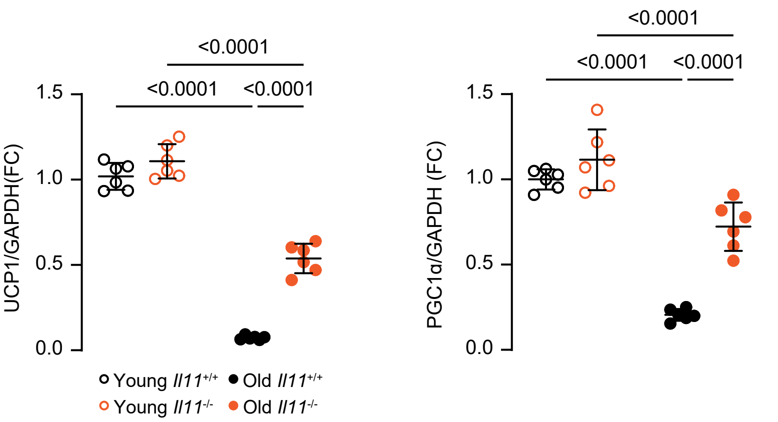

Extended Data Fig 1b

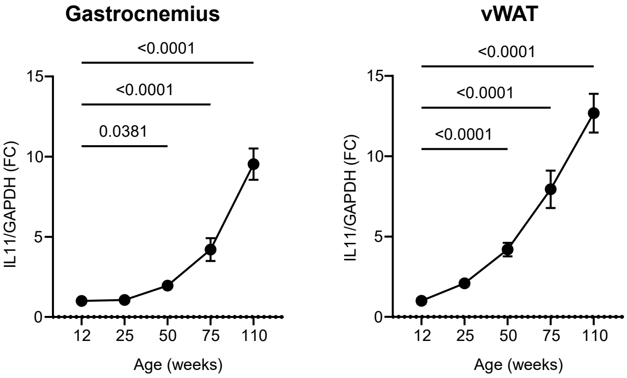

Extended Data Fig 1c

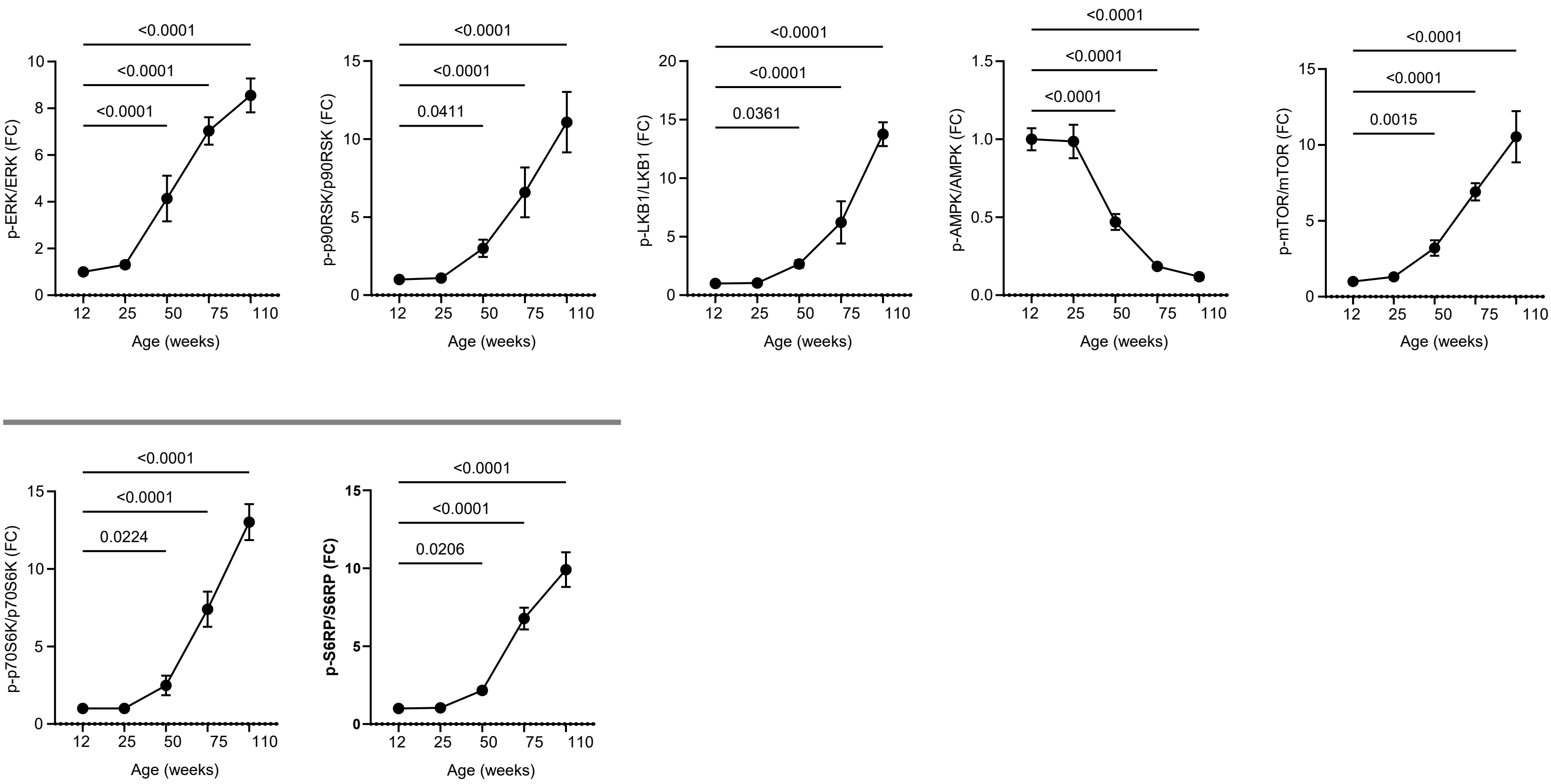

Extended Data Fig 1d

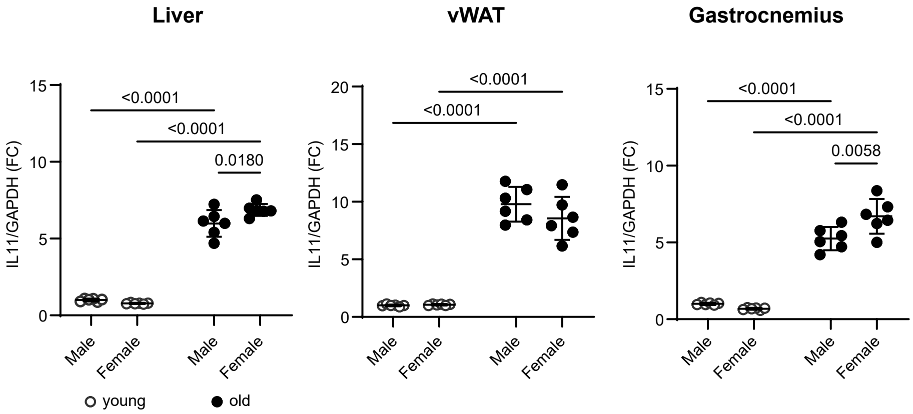

Extended Data Fig 2b

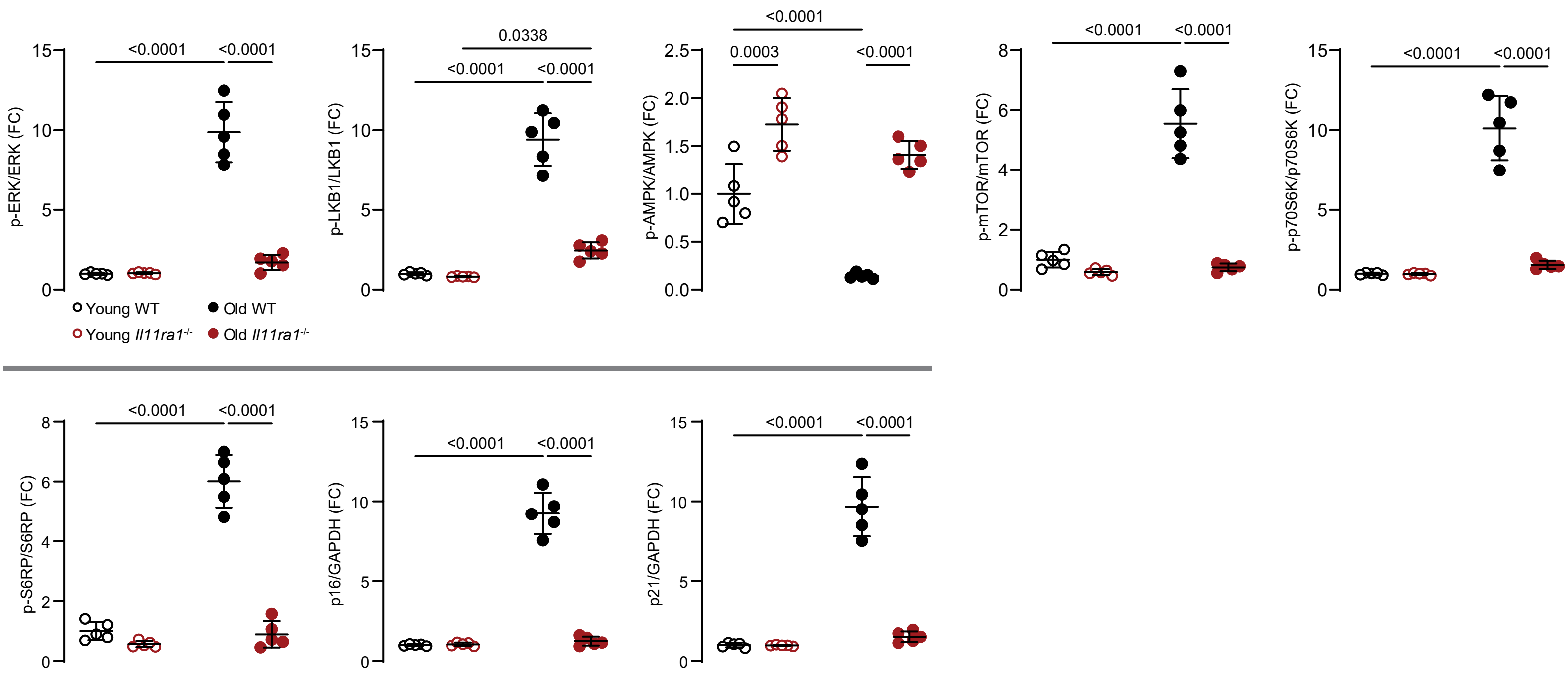

Extended Data Fig 2c

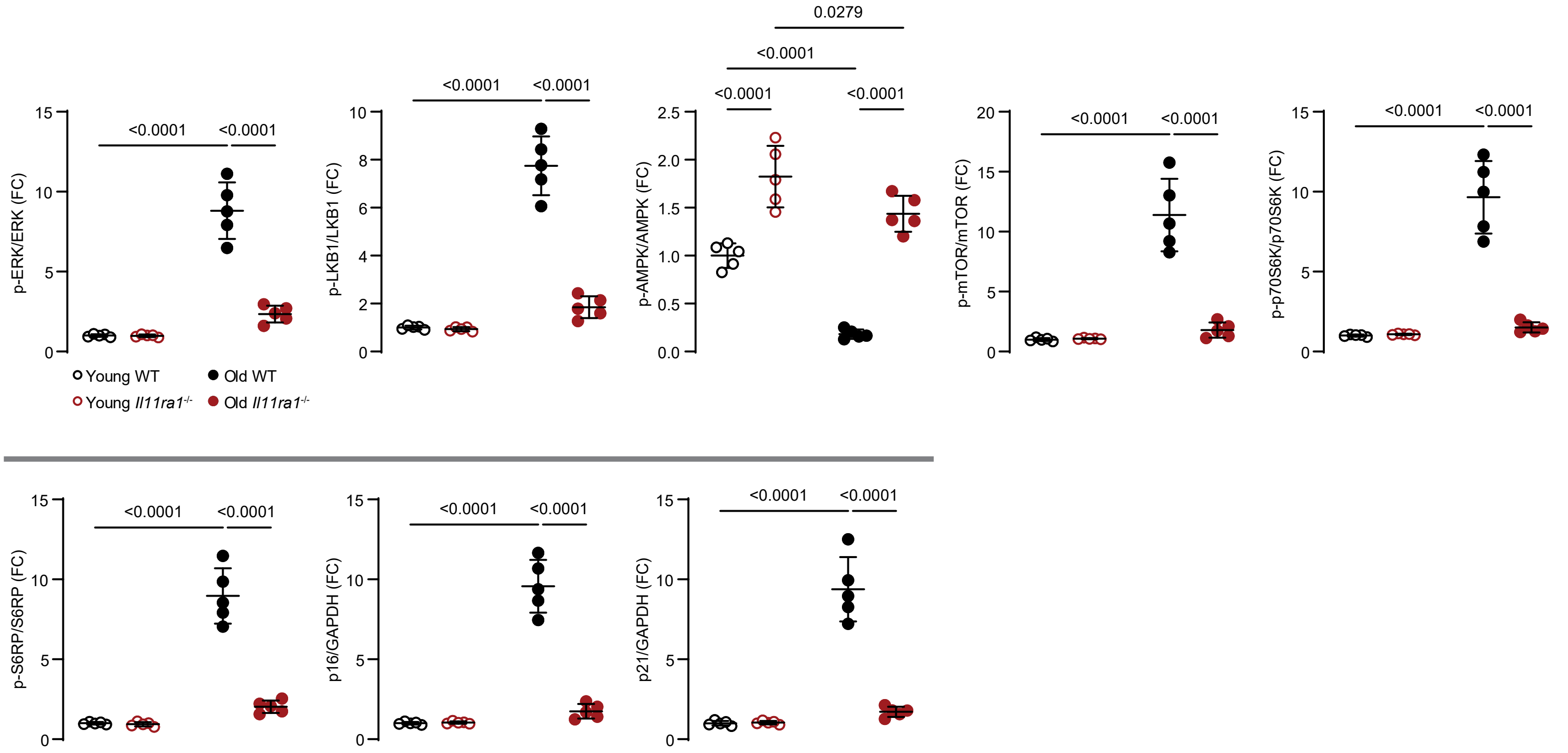

Extended Data Fig 3a

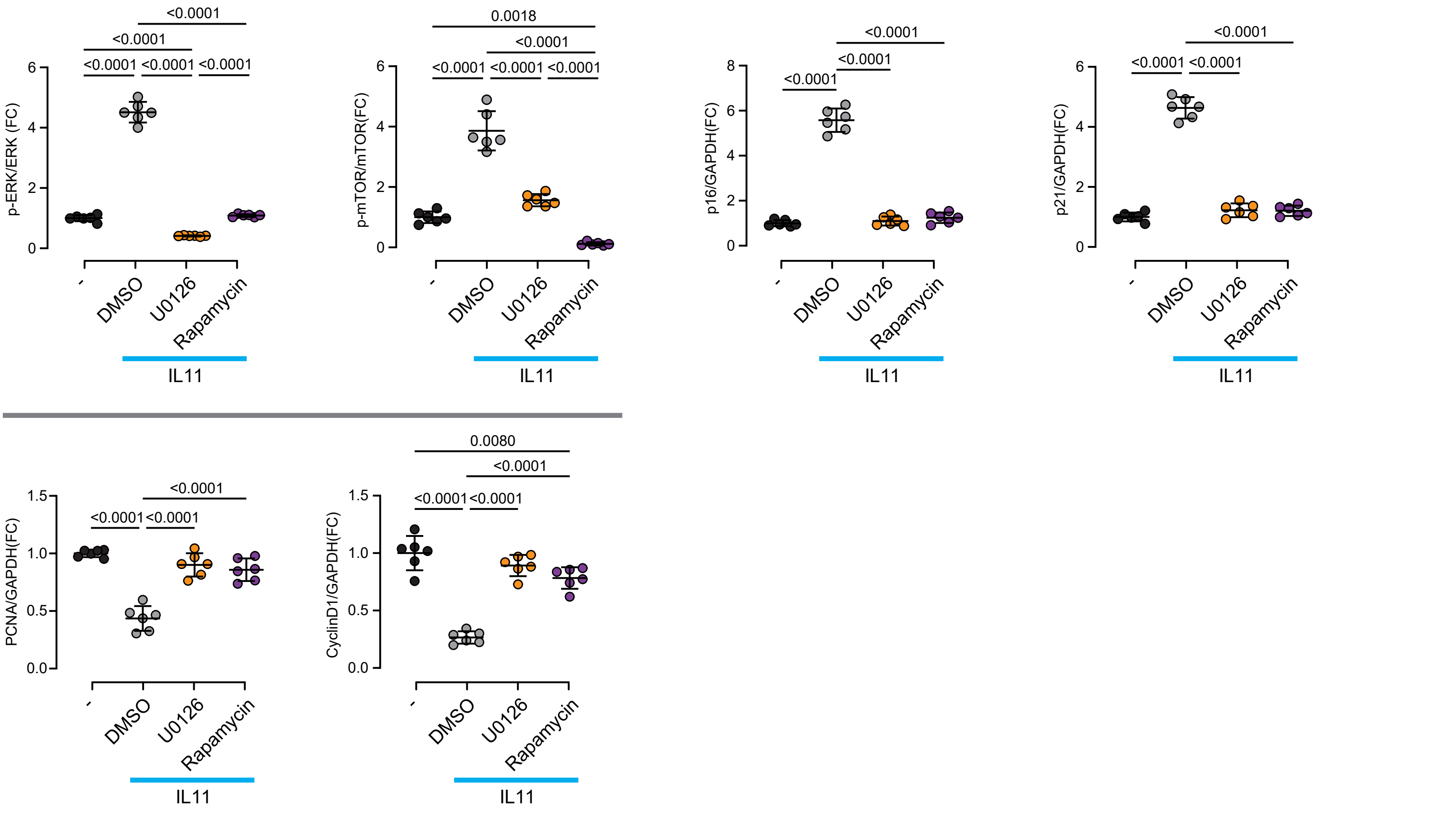

Extended Data Fig 3b

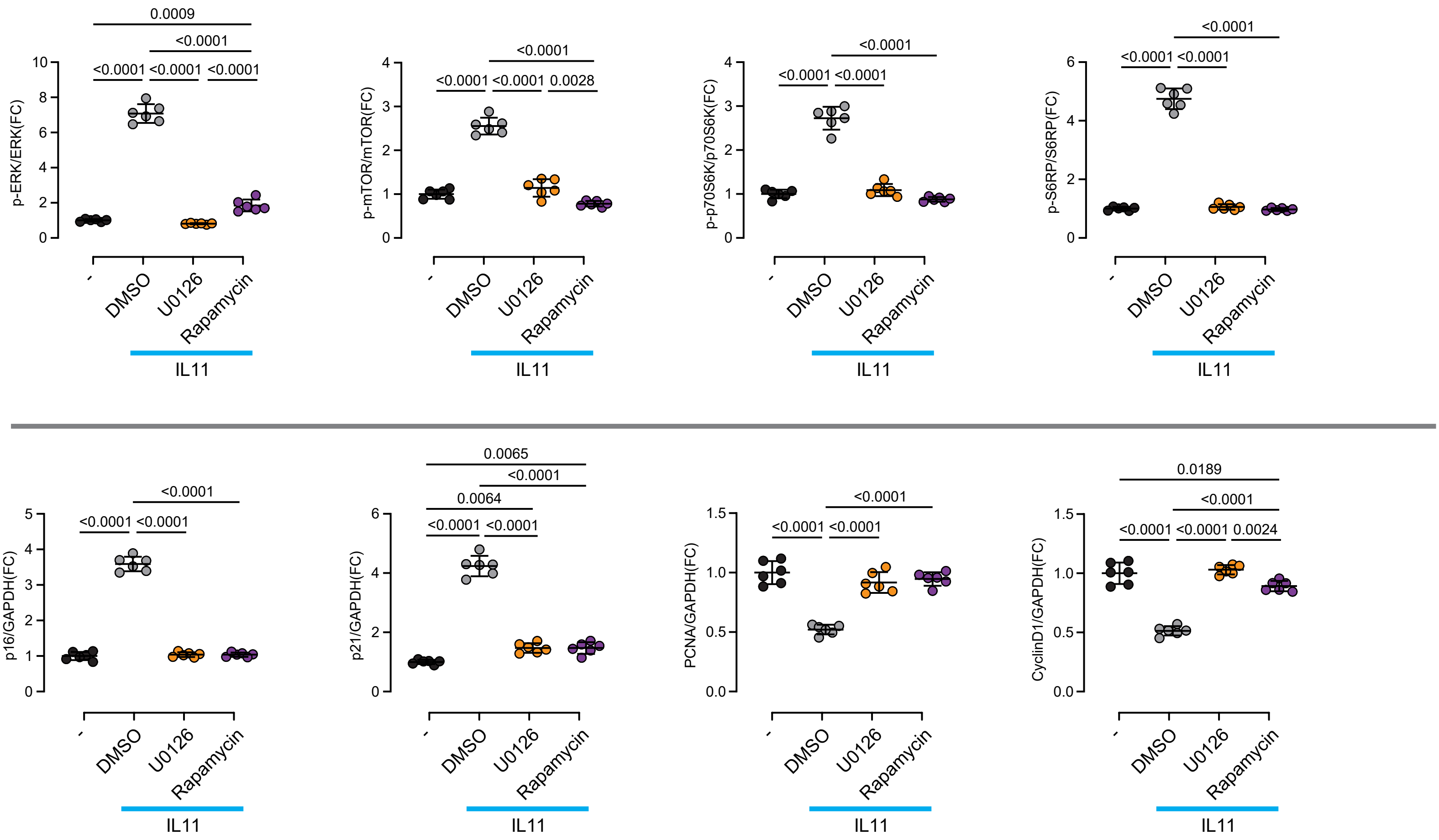

Extended Data Fig 4a

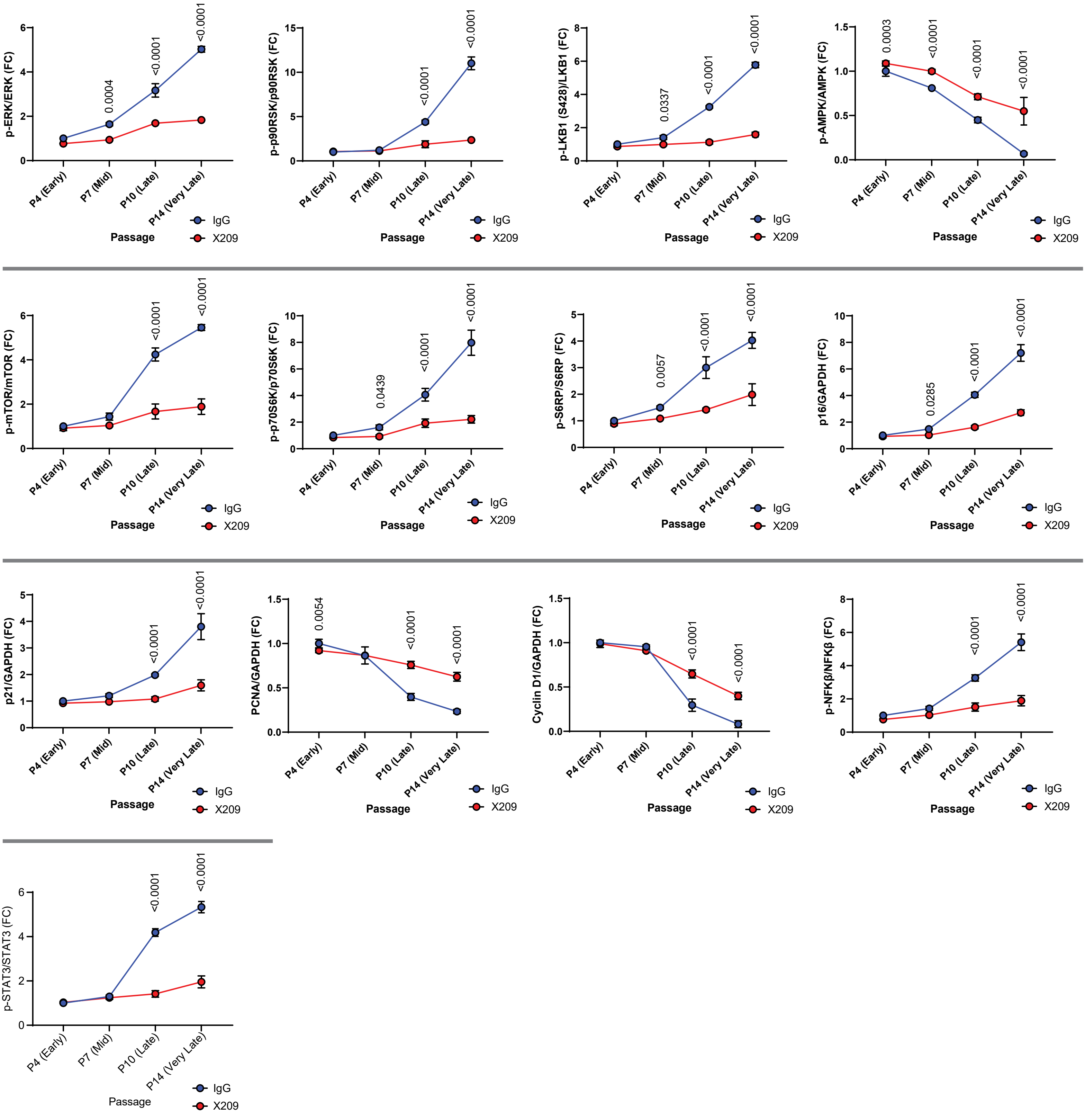

Extended Data Fig 4d

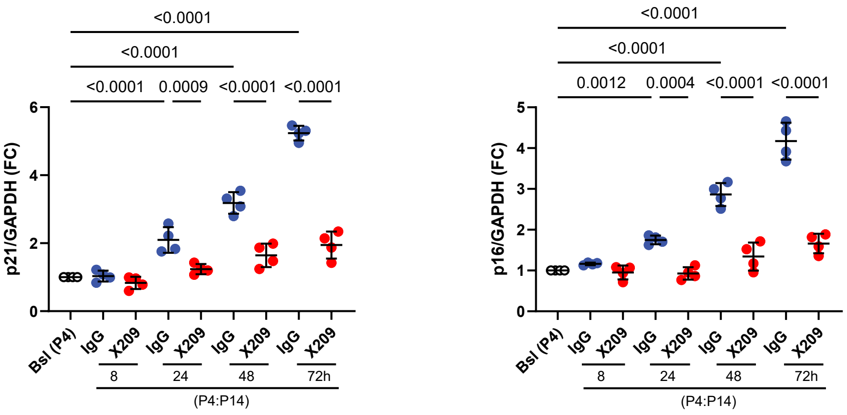

Extended Data Figure 5l

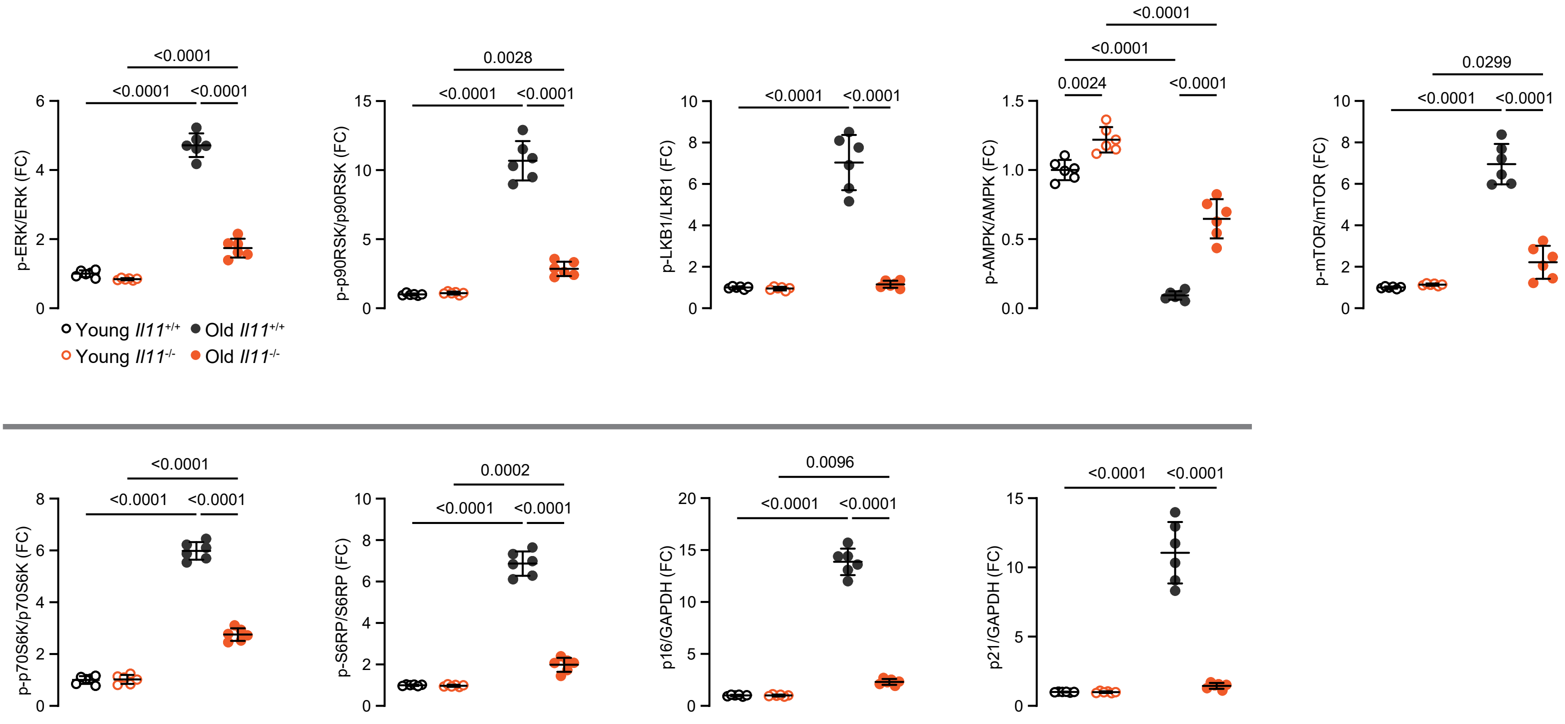

Supplement: Supplementary file 1 — Supplementary Figs. 1 and 2, which include the raw blots and immunoblot densitometry analysis. [file 41586_2024_7701_MOESM1_ESM.pdf]
