# Supplementary material for: Inhibition of IL-11 signalling extends mammalian healthspan and lifespan
Source: Nature. 2024 Jul 17;632(8023):157–65. doi: 10.1038/s41586-024-07701-9 (PMC11291288; doi:10.1038/s41586-024-07701-9)
Supplement: Supplementary file 2 — Reporting Summary [file 41586_2024_7701_MOESM2_ESM.pdf]

nature portfolio

Corresponding author(s): Anissa A Widjaja and Stuart A Cook

Last updated by author(s): May 21, 2024

Reporting Summary

Nature Portfolio wishes to improve the reproducibility of the work that we publish. This form provides structure for consistency and transparency in reporting. For further information on Nature Portfolio policies, see our Editorial Policies and the Editorial Policy Checklist.

Statistics

For all statistical analyses, confirm that the following items are present in the figure legend, table legend, main text, or Methods section.

n/a	Confirmed	
☐	☒	The exact sample size (*n*) for each experimental group/condition, given as a discrete number and unit of measurement
☐	☒	A statement on whether measurements were taken from distinct samples or whether the same sample was measured repeatedly
☐	☒	The statistical test(s) used AND whether they are one- or two-sided<br>*Only common tests should be described solely by name; describe more complex techniques in the Methods section.*
☒	☐	A description of all covariates tested
☐	☒	A description of any assumptions or corrections, such as tests of normality and adjustment for multiple comparisons
☐	☒	A full description of the statistical parameters including central tendency (e.g. means) or other basic estimates (e.g. regression coefficient) AND variation (e.g. standard deviation) or associated estimates of uncertainty (e.g. confidence intervals)
☐	☒	For null hypothesis testing, the test statistic (e.g. *F*, *t*, *r*) with confidence intervals, effect sizes, degrees of freedom and *P* value noted<br>*Give P values as exact values whenever suitable.*
☒	☐	For Bayesian analysis, information on the choice of priors and Markov chain Monte Carlo settings
☒	☐	For hierarchical and complex designs, identification of the appropriate level for tests and full reporting of outcomes
☒	☐	Estimates of effect sizes (e.g. Cohen's *d*, Pearson's *r*), indicating how they were calculated

Our web collection on statistics for biologists contains articles on many of the points above.

Software and code

Policy information about availability of computer code

Data collection	The study does not involve software and code for data collection.
Data analysis	GraphPad Prism (version 10), ImageJ (version 1.53f51), bcl2fastq (v2.20.0.422), Trimmomatic (V0.36), STAR (V2.7.9), DESeq2 (V1.36.0), fgsea R package (v.1.22.0)

For manuscripts utilizing custom algorithms or software that are central to the research but not yet described in published literature, software must be made available to editors and reviewers. We strongly encourage code deposition in a community repository (e.g. GitHub). See the Nature Portfolio guidelines for submitting code & software for further information.

Data

Policy information about availability of data

All manuscripts must include a data availability statement. This statement should provide the following information, where applicable:

- Accession codes, unique identifiers, or web links for publicly available datasets
- A description of any restrictions on data availability
- For clinical datasets or third party data, please ensure that the statement adheres to our policy

All data are available within the Article or Supplementary Information. The RNAseq data reported in this paper are available on the Short Read Archive with Bioproject ID: PRJNA939262 (https://www.ncbi.nlm.nih.gov/bioproject/PRJNA939262). Datasets used for analysis in this study are as follows: Ensembl release 104 Mus musculus GRCm39 gene annotations (GRCm39, https://asia.ensembl.org/info/data/ftp/index.html), MSigDB Hallmark (v.7.5.1, https://www.gsea-msigdb.org/

gsea/msigdb/human/collections.jsp), MitoCarta (v3.0, https://www.broadinstitute.org/mitocarta/mitocarta30-inventory-mammalian-mitochondrial-proteins-and-pathways). Source data are provided with this paper

Human research participants

Policy information about studies involving human research participants and Sex and Gender in Research.

Reporting on sex and gender	The study did not involve human research participants
Population characteristics	N/A
Recruitment	N/A
Ethics oversight	N/A

Note that full information on the approval of the study protocol must also be provided in the manuscript.

Field-specific reporting

Please select the one below that is the best fit for your research. If you are not sure, read the appropriate sections before making your selection.

☒ Life sciences ☐ Behavioural & social sciences ☐ Ecological, evolutionary & environmental sciences

For a reference copy of the document with all sections, see nature.com/documents/nr-reporting-summary-flat.pdf

Life sciences study design

All studies must disclose on these points even when the disclosure is negative.

Sample size	Sample sizes were determined based on the authors' experience with the preliminary in vitro, in vivo, and molecular studies and by referencing a healthspan/lifespan study in mice by Ackert-Bicknell et al (PMID: 26069080) to detect a 20% change in phenotype between treatment groups or genotypes with 80% power (α=0.05).<br>Sample sizes for experiments involving Il11ra1-/- and Il11-/- mice (and their respective WT mice) varied depending on animal availability.<br>Sample size for cell based assays were determined based on sample availability.
Data exclusions	Outlier tests were conducted using the ROUT method in GraphPad Prism, with the maximum false discovery rate (FDR) set to 1%.
Replication	All experiments were repeated with reproducibility. The replication number for each experiment is indicated in the legend of the corresponding figure.
Randomization	For in vitro studies, equal number of cells were seeded and allocated randomly into experimental groups. For in vivo studies, mice were randomly allocated to experimental groups on the day of the treatment except for Il11ra1-/- and Il11-/- in which randomization was not applicable.
Blinding	For in vitro experiments, investigators were not blinded to group allocation during data collection and analysis as they were performed by a single individual. For in vivo experiments, treatments/genotypes were not disclosed to investigators generating quantitative readouts during data collection but were revealed during the analysis. Histological analysis were performed blinded to treatments and genotypes.

Reporting for specific materials, systems and methods

We require information from authors about some types of materials, experimental systems and methods used in many studies. Here, indicate whether each material, system or method listed is relevant to your study. If you are not sure if a list item applies to your research, read the appropriate section before selecting a response.

Materials & experimental systems

n/a	Involved in the study	
☐	☒	Antibodies
☐	☒	Eukaryotic cell lines
☒	☐	Palaeontology and archaeology
☐	☒	Animals and other organisms
☒	☐	Clinical data
☒	☐	Dual use research of concern

Methods

n/a	Involved in the study	
☒	☐	ChIP-seq
☒	☐	Flow cytometry
☒	☐	MRI-based neuroimaging

Antibodies

Antibodies used

For all the commercially available antibodies used in this study, the recommended dilutions from the manufacturer's instructions have been followed

The following commercially available primary antibodies were used for Western Blot. They are listed as antigen followed by catalog number, clone name (for monoclonal antibody), supplier name, and dilution ratio:
1. p-AMPK, 2535, clone 40H9, CST, 1:1000.
2. AMPK, 5832, clone D63G4, CST, 1:1000.
3. Cyclin D1, 55506, clone E3P5S, CST, 1:1000.
4. p-ERK1/2, 4370, clone D13.14.4E, CST, 1:1000.
5. ERK1/2, 4695, clone 137F5, CST, 1:1000.
6. GAPDH 2118, clone 14C10, CST, 1:1000.
7. IL11, NA, clone X203, Aldevron, 1:1000.
8. p-LKB1, 3482, clone C67A3, CST, 1:1000.
9. LKB1, 3047, clone D60C5, CST, 1:1000.
10. p-mTOR, 2971, NA, CST, 1:1000.
11. mTOR, 2972, NA, CST, 1:1000.
12. p-NFκB, 3033, clone 93H1, CST, 1:1000.
13. NFκB, 8242, clone D14E12, CST, 1:1000.
14. Human p16, ab108349, clone EPR1473, abcam, 1:1000.
15. Mouse p16, ab232402, clone EPR20418, abcam, 1:1000.
16. Human p21, ab109520, clone EPR362 abcam, 1:1000.
17. Mouse p21, ab188224, clone EPR18021, abcam, 1:1000.
18. p-p70S6K, 9234, clone 108D2, CST, 1:1000.
19. p70S6K, 2708, clone 49D7, CST, 1:1000.
20. p-p90RSK, 11989, clone D3H11, CST, 1:1000.
21. p90RSK, 9355, clone 32D7, CST, 1:1000.
22. PCNA, 13110, clone D3H8P, CST, 1:1000.
23. PGC1a, ab191838, NA, abcam, 1:1000.
24. p-S6RP, 4858, clone D57.2.2E, CST, 1:1000.
25. S6RP, 2217, clone 5G10, CST, 1:1000.
26. pSTAT3, 4113, clone M9C6, CST, 1:1000.
27. STAT3, 4904, clone 79D7, CST, 1:1000.
28. UCP1, 72298, clone E9Z2V, CST, 1:1000.
29. HRP conjugated anti-mouse IgG (H+L)/anti-mouse HRP, 7076, NA, CST, 1:1000.
30. HRP conjugated anti-rabbit IgG (H+L)/anti-rabbit HRP, 7074, NA, CST, 1:1000.

The following custom-made primary antibodies were used for neutralization study (in vitro/in vivo treatment). They are listed as antigen followed by catalog number, clone name (for monoclonal antibody), supplier name, and dilution ratio:
1. IgG, NA, clone 11E10, Aldevron, in vitro: 2 µg/ml, in vivo: 40 mg/kg
2. IL11, NA, clone X203, Aldevron, in vitro: 2 µg/ml, in vivo: 40 mg/kg
3. IL11RA, NA, clone X209, Aldevron, in vitro: 2 µg/ml.

The following commercially available primary antibodies were used for Operetta phenotyping assay. They are listed as antigen followed by catalog number, clone name (for monoclonal antibody), supplier name, and dilution ratio:
1. Human p16, ab108349, clone EPR1473, abcam, 1:1000.
2. Human p21, ab109520, clone EPR362 abcam, 1:1000.
3. Anti-rabbit Alexa Fluor 488, ab150077, NA, abcam, 1:1000.

The following commercially available primary antibodies were used for immunofluoresence. They are listed as antigen followed by catalog number, clone name (for monoclonal antibody), supplier name, and dilution ratio:
1. Adiponectin, 21613-1-AP, NA, Proteintech, 1:100
2. CD31, ab222783, clone EPR17260-263, abcam, 1:200
3. FHL1, 10991-1-AP, NA, Proteintech, 1:200
4. GFP, ab290, NA, abcam, 1:100
5. GFP, ab6673, NA, abcam, 1:100
6. PDGFRa, AF1062, NA, R&D systems, 1:100
7. SLC10A1, MBS177905, NA, MyBioSource, 1:100
8. SM22a, ab14106, NA, abcam, 1:200
9. Anti-rabbit Alexa Fluor 488, ab150077, NA, abcam, 1:300
10. Anti-goat Alexa Fluor 488, ab150129, NA, abcam, 1:300
11. Anti-rabbit Alexa Fluor 555, ab150074, NA, abcam, 1:300

The following commercially available primary antibody was used for immunohistochemistry. It is listed as antigen followed by catalog number, clone name (for monoclonal antibody), supplier name, and dilution ratio:
1. CD68, ab125212, NA, abcam, 1:100.

Validation

All commercially available antibodies have been validated by the manufacturers for the applications used in this study as indicated on the respective manufacturer's website. The custom-made antibodies i.e: IgG (11E10), anti-IL11 (X203), and anti-IL11RA (X209) were validated by citations. Manufacturer's website containing validation data (also listed here) for the commercially available antibodies and citations for the custom-made antibodies are listed below:

1. Adiponectin (https://www.ptglab.com/products/ADIPOQ-Antibody-21613-1-AP.htm); species reactivity: mouse; validated application: IF (in NIH/3T3 cells).
2. p-AMPK (https://www.cellsignal.com/products/primary-antibodies/phospho-ampka-thr172-40h9-rabbit-mab/2535); species reactivity: human, mouse; validated application: WB (using 293T (human) and C2C12 (mouse) cell lysates).
3. AMPK (https://www.cellsignal.com/products/primary-antibodies/ampka-d63g4-rabbit-mab/5832); species reactivity: human, mouse; validated application: WB (using HeLa, K-562 (human) and Neuro-2a (mouse) cell lysates).
4. CD31 (https://www.abcam.com/products/primary-antibodies/cd31-antibody-epr17260-263-ab222783.html); species reactivity: mouse; validated application: IF (on bEND.3 and NIH/3T3 cells).
5. CD68 (https://www.abcam.com/products/primary-antibodies/cd68-antibody-ab125212.html); species reactivity: mouse; validated application: IHC (on paraformaldehyde-fixed, paraffin-embedded mouse brain, skin, spleen, and liver).
6. Cyclin D1 (https://www.cellsignal.com/products/primary-antibodies/cyclin-d1-e3p5s-xp-rabbit-mab/55506); species reactivity: human, mouse; validated application: WB (using SH-SY5Y cell (human) and mouse liver and kidney lysates).
7. p-ERK1/2 (https://www.cellsignal.com/products/primary-antibodies/phospho-p44-42-mapk-erk1-2-thr202-tyr204-d13-14-4e-xp-rabbit-mab/4370); species reactivity: human, mouse; validated application: WB (using 293 (human) and NIH/3T3 (mouse) cell lysates).
8. ERK1/2 (https://www.cellsignal.com/products/primary-antibodies/p44-42-mapk-erk1-2-137f5-rabbit-mab/4695); species reactivity: human, mouse; validated application: WB (using HeLa (human) and NIH/3T3 (mouse) cell lysates).
9. FHL1 (https://www.ptglab.com/products/FHL1-Antibody-10991-1-AP.htm); species reactivity: mouse; this antibody is not validated for IF but it's listed as the suitable application - validated application: WB (using mouse skeletal muscle lysates).
10. GAPDH (https://www.cellsignal.com/products/primary-antibodies/gapdh-14c10-rabbit-mab/2118); species reactivity: human, mouse; validated application: WB (using HeLa (human) and NIH/3T3 (mouse) cell lysates).
11. GFP (ab290) (https://www.abcam.com/products/primary-antibodies/gfp-antibody-ab290.html); species reactivity: mouse; validated application: IF (using GFP-transfected NIH/3T3 cells).
12. GFP (ab6673) (https://www.abcam.com/products/primary-antibodies/gfp-antibody-ab6673.html); species reactivity: mouse; validated application: IF (in Hex-GFP transgenic mouse embryo se).
13. IgG (11E10) was validated for its non-ability to have any effect on human/mouse cells in PMID: 34108253.
14. IL11 (X203) was validated for neutralization of human and mouse IL11 in PMID: 31554736 and for WB (mouse liver lysates) in PMID: 31078624; species reactivity: human and mouse.
15. IL11RA (X209) was validated for neutralization of human IL11RA in PMID: 31078624, 34108253, 36470928; species reactivity: human.
16. p-LKB1 (https://www.cellsignal.com/products/primary-antibodies/phospho-lkb1-ser428-c67a3-rabbit-mab/3482); species reactivity: human, mouse; validated application: WB (using Caki (human) and L929 (mouse) cell lysates).
17. LKB1 (https://www.cellsignal.com/products/primary-antibodies/lkb1-d60c5-rabbit-mab/3047); species reactivity: human, mouse; validated application: WB (using Caki (human) and L929 (mouse) cell lysates).
18. p-mTOR (https://www.cellsignal.com/products/primary-antibodies/phospho-mtor-ser2448-antibody/2971); species reactivity: human, mouse; validated application: WB (using 293, MCF-7 cell (human) and mouse liver, heart, skeletal muscle lysates).
19. mTOR (https://www.cellsignal.com/products/primary-antibodies/mtor-antibody/2972); species reactivity: human, mouse; validated application: WB (using 293, HeLa (human) and C2C12 (mouse) cell lysates).
20. p-NFκB (https://www.cellsignal.com/products/primary-antibodies/phospho-nf-kb-p65-ser536-93h1-rabbit-mab/3033); species reactivity: human; validated application: WB (using HeLa cell lysates).
21. NFκB (https://www.cellsignal.com/products/primary-antibodies/nf-kb-p65-d14e12-xp-rabbit-mab/8242); species reactivity: human; validated application: WB (using HeLa, MCF-7 cell lysates).
22. p16 (https://www.abcam.com/products/primary-antibodies/cdkn2ap16ink4a-antibody-epr1473-c-terminal-ab108349.html); species reactivity: human; validated application: WB (using HeLa cell lysates), this antibody is not validated for IF but it's listed as one of the possible applications.
23. p16 (https://www.abcam.com/products/primary-antibodies/cdkn2ap16ink4a-antibody-epr20418-bsa-and-azide-free-ab232402.html); species reactivity: mouse; validated application: WB (using MEF cell lysates).
24. p21 (https://www.abcam.com/products/primary-antibodies/p21-antibody-epr362-ab109520.html); species reactivity: human; validated application: WB (using HeLa cell lysates), IF (in MCF-7 cells).
25. p21 (https://www.abcam.com/products/primary-antibodies/p21-antibody-epr18021-ab188224.html); species reactivity: mouse; validated application: WB (using NIH/3TC cell lysates).
26. p-p70S6K (https://www.cellsignal.com/products/primary-antibodies/phospho-p70-s6-kinase-thr389-108d2-rabbit-mab/9234); species reactivity: human, mouse; validated application: WB (using 293T (human) and NIH/3T3 (mouse) cell lysates).
27. p70S6K (https://www.cellsignal.com/products/primary-antibodies/p70-s6-kinase-49d7-rabbit-mab/2708); species reactivity: human, mouse; validated application: WB (using HeLa, MCF-7 cell (human) and mouse kidney, liver, skeletal muscle, heart, adipocyte lysates).
28. p-p90RSK (https://www.cellsignal.com/products/primary-antibodies/phospho-p90rsk-ser380-d3h11-rabbit-mab/11989); species reactivity: human, mouse; validated application: WB (using HeLa (human) and NIH/3T3 (mouse) cell lysates).
29. p90RSK (https://www.cellsignal.com/products/primary-antibodies/rsk1-rsk2-rsk3-32d7-rabbit-mab/9355); species reactivity: human, mouse; validated application: WB (using HeLa (human) and L929 (mouse) cell lysates).
30. PCNA (https://www.cellsignal.com/products/primary-antibodies/pcna-d3h8p-xp-rabbit-mab/13110); species reactivity: human; validated application: WB (using HeLa, MCF-7 cell lysates).
31. PDGFRa (https://www.rndsystems.com/products/mouse-pdgf-ralpha-antibody_af1062); species reactivity: mouse; validated application: IF (on mouse intestinal villus tip telocytes).
32. PGC1a (https://www.abcam.com/products/primary-antibodies/pgc1-alpha-antibody-n-terminal-ab191838.html); species reactivity: mouse; validated application: WB (using mouse skin lysates).
33. p-S6RP (https://www.cellsignal.com/products/primary-antibodies/phospho-s6-ribosomal-protein-ser235-236-d57-2-2e-xp-rabbit-mab/4858); species reactivity: human, mouse; validated application: WB (using MCF-7 cell (human) and mouse liver, skeletal muscle, heart lysates).
34. S6RP (https://www.cellsignal.com/products/primary-antibodies/s6-ribosomal-protein-5g10-rabbit-mab/2217); species reactivity: human, mouse; validated application: WB (using HeLa (human) and NIH/3T3 (mouse) cell lysates).
35. SLC10A1 (https://www.mybiosource.com/polyclonal-mouse-rat-antibody/slc10a1/177905); species reactivity: mouse; validated application: IF (on paraffin-embedded section of mouse liver tissues).
36. SM22a (https://www.abcam.com/products/primary-antibodies/taglntransgelin-antibody-ab14106.html); species reactivity: mouse; validated application: IF (on mouse muscle cells).
37. p-STAT3 (https://www.cellsignal.com/products/primary-antibodies/phospho-stat3-tyr705-m9c6-mouse-mab/4113); species

reactivity: human; validated application: WB (using HeLa cell lysates).
38. STAT3 (https://www.cellsignal.com/products/primary-antibodies/stat3-79d7-rabbit-mab/4904); species reactivity: human; validated application: WB (using HeLa cell lysates).
39. UCP1 (https://www.cellsignal.com/products/primary-antibodies/ucp1-e9z2v-xp-rabbit-mab/72298); species reactivity: mouse; validated application: WB (using mouse brown adipose tissues)
40. Anti-rabbit HRP (https://www.cellsignal.com/products/secondary-antibodies/anti-rabbit-igg-hrp-linked-antibody/7074); designed for use with rabbit polyclonal and monoclonal antibodies, this affinity purified goat anti-rabbit IgG (heavy and light chain) antibody is conjugated to horseradish peroxidase (HRP) for chemiluminescent detection; validated application: WB (This product is thoroughly validated with CST primary antibodies).
41. Anti-mouse HRP (https://www.cellsignal.com/products/secondary-antibodies/anti-mouse-igg-hrp-linked-antibody/7076); affinity purified horse anti-mouse IgG (heavy and light chain) antibody is conjugated to HRP for chemiluminescent detection; validated application: WB (This product is thoroughly validated with CST primary antibodies).
42. Goat anti-rabbit Alexa Fluor 488 (https://www.abcam.com/goat-rabbit-igg-hl-alexa-fluor-488-ab150077.html); validated application: IF.
43. Donkey anti-goat Alexa Fluor 488 (https://www.abcam.com/donkey-goat-igg-hl-alexa-fluor-488-ab150129.html); validated application: IF.
44. Donkey anti-goat Alexa Fluor 555(https://www.abcam.com/products/secondary-antibodies/donkey-rabbit-igg-hl-alexa-fluor-555-ab150074.html); validated application: IF.

Eukaryotic cell lines

Policy information about cell lines and Sex and Gender in Research

Cell line source(s)

Primary human cardiac fibroblasts (HCFs, 52-year-old male, 6330, lot #9580, ScienCell).
Primary human hepatocytes were isolated from a 22-week-old foetus (5200, lot #34967, ScienCell).

Authentication

Primary human cardiac fibroblasts (HCFs) and primary human hepatocytes were authenticated by ScienCell based on their morphology and by using immunofluorescence (IF) for cell-specific markers, as detailed in the respective product datasheet and certificate of analysis (COA).

Primary HCFs were authenticated by their fibroblast morphology and phenotype, characterised by IF staining for fibronectin and vimentin.

Primary human hepatocytes were authenticated by their hepatocyte morphology and phenotype, characterised by positive IF for cytokeratin-18 and WB for albumin.

Mycoplasma contamination

All cell lines were tested to be free of mycoplasma contamination.

Commonly misidentified lines
(See ICLAC register)

No commonly misidentified cell lines were used in the study.

Animals and other research organisms

Policy information about studies involving animals; ARRIVE guidelines recommended for reporting animal research, and Sex and Gender in Research

Laboratory animals

All mice were housed at 21-24? with 40-70% humidity on a 12-hour light/dark cycle and provided food and water ad libitum. Our mouse colonies hold specific pathogen free (SPF) status and undergo quarterly and annual tests for common pathogens. The room housing our animals is Murine Norovirus and Helicobacter positive, and these particular pathogens are deemed acceptable within our SPF facility. The mouse strains used in our study are as follow:
1. Il11ra1-deleted mice (Il11ra1−/− or Il11ra1 KO)
Male and female Il11ra1+/+ (wild-type) and Il11ra1-/- mice25 (B6.129S1-Il11ratm1Wehi/J, The Jackson Laboratory) were sacrificed at 110 weeks of age for blood and tissue collection; 10-12 weeks old male and female mice of the respective genotypes were used as controls.
2. Il11-deleted mice (Il11−/−)
Male and female mice lacking functional alleles for Il11 (Il11−/−), which were generated and characterised previously31,51, and their wild-type counterparts were sacrificed at 10-12 weeks of age (young controls) and 104-108 weeks of age (old mice).
3. Il11-EGFP reporter mice
Young (10-week-old) and old (100-week-old) transgenic mice (C57BL/6J background) with EGFP knocked-into the Il11 gene (Il11-EGFP mice, Cyagen Biosciences Inc)29 were sacrificed for IF staining studies of liver, gastrocnemius, and visceral gonadal white adipose tissue (referred in the text as vWAT). Old wild-type littermates were used as aged negative controls.
4. In vivo administration of anti-IL11
Male and female C57BL/6J mice (Jackson Laboratory) were randomised prior to receiving either no treatment, anti-IL11 (X203) or IgG (11E10). X203 or 11E10 (40 mg/kg, every 3 weeks) were administered by intraperitoneal injection, starting from 75 weeks of age for a duration of 25 weeks; mice were then sacrificed at 100 weeks of age.

Wild animals

The study does not involve wild animals.

Reporting on sex	This study use both male and female mice.
Field-collected samples	The study does not involve field collected animals.
Ethics oversight	Animal studies were carried out in compliance with the recommendations in the Guidelines on the Care and Use of Animals for Scientific Purposes of the National Advisory Committee for Laboratory Animal Research (NACLAR). All experimental procedures were approved (SHS/2019/1481 and SHS/2019/1483) and conducted in accordance with the SingHealth Institutional Animal Care and Use Committee (IACUC). Certified veterinarians were responsible for all animal experiment procedures according to the laws governing animal research in Singapore.

Note that full information on the approval of the study protocol must also be provided in the manuscript.
