# Supplementary material for: Inhibition of IL-11 signalling extends mammalian healthspan and lifespan
Source: Nature. 2024 Jul 17;632(8023):157–65. doi: 10.1038/s41586-024-07701-9 (PMC11291288; doi:10.1038/s41586-024-07701-9)
Supplement: Supplementary file 10 — Primer list. [file 41586_2024_7701_MOESM10_ESM.pdf]

Extended Data Table 8. Primer list.

Host	Gene	Forward (5'-3')	Reverse (5'-3')
Mouse	***Acc1***	ATTGGGCACCCAGAGCTA	CCCGCTCCTTCAACTTGCT
	Ccl2	GAAGGAATGGGTCCAGACAT	ACGGGTCAACTTCACATTCA
	Ccl5	GCTGCTTTGCCTACCTCTCC	TCGAGTGACAAACACGACTGC
	Fasn	CATGACCTCGTGATGAACGTGT	CGGGTGAGGACGTTTACAAAG
	Gapdh	CTGGAAAGCTGTGGCGTGAT	GACGGACACATTGGGGGTAG
	Il1β	CACAGCAGCACATCAACAAG	GTGCTCATGTCCTCATCCTG
	Il6	CTCTGGGAAATCGTGGAAAT	CCAGTTTGGTAGCATCCATC
	Srebp1c	ATCGGCGCGGAAGCTGTCGGGGT	ACTGTCTTGGTTGTTGATGAGCT
	Tnfα	ATGAGAAGTTCCCAAATGGC	CTCCACTTGGTGGTTTGCTA
	Ucp1	ACTGCCACACCTCCAGTCATT	CTTTGCCTCACTCAGGATTGG
